# *Trans*-ancestry Fine Mapping and Molecular Assays Identify Regulatory Variants at the *ANGPTL8* HDL-C GWAS Locus

**DOI:** 10.1534/g3.117.300088

**Published:** 2017-07-28

**Authors:** Maren E. Cannon, Qing Duan, Ying Wu, Monica Zeynalzadeh, Zheng Xu, Antti J. Kangas, Pasi Soininen, Mika Ala-Korpela, Mete Civelek, Aldons J. Lusis, Johanna Kuusisto, Francis S. Collins, Michael Boehnke, Hua Tang, Markku Laakso, Yun Li, Karen L. Mohlke

**Affiliations:** * Department of Genetics, University of North Carolina, Chapel Hill, North Carolina 27599; † Computational Medicine, Institute of Health Sciences, University of Oulu and Biocenter Oulu, 90014 Finland; ‡ NMR Metabolomics Laboratory, School of Pharmacy, University of Eastern Finland, Kuopio, 70600 Finland; § School of Social and Community Medicine, University of Bristol and Medical Research Council Integrative Epidemiology Unit at the University of Bristol, BS8 1TH United Kingdom; ** Center for Public Health Genomics and Department of Biomedical Engineering, University of Virginia, Charlottesville, Virginia 22904; †† Department of Medicine, Department of Human Genetics, Molecular Biology Institute, Department of Microbiology, Immunology and Molecular Genetics, University of California, Los Angeles, California 90095; ‡‡ Institute of Clinical Medicine, Internal Medicine, University of Eastern Finland and Kuopio University Hospital, 70600 Finland; §§ National Human Genome Research Institute, National Institutes of Health, Bethesda, Maryland 20892; *** Department of Biostatistics and Center for Statistical Genetics, University of Michigan, Ann Arbor, Michigan 48109; ††† Department of Genetics, Stanford University, California 94305; ‡‡‡ Department of Biostatistics, University of North Carolina, Chapel Hill, North Carolina 27599

**Keywords:** Genetics, Gene Expression, Cholesterol, Transcription, Complex genetic traits

## Abstract

Recent genome-wide association studies (GWAS) have identified variants associated with high-density lipoprotein cholesterol (HDL-C) located in or near the *ANGPTL8* gene. Given the extensive sharing of GWAS loci across populations, we hypothesized that at least one shared variant at this locus affects HDL-C. The HDL-C–associated variants are coincident with expression quantitative trait loci for *ANGPTL8* and *DOCK6* in subcutaneous adipose tissue; however, only *ANGPTL8* expression levels are associated with HDL-C levels. We identified a 400-bp promoter region of *ANGPTL8* and enhancer regions within 5 kb that contribute to regulating expression in liver and adipose. To identify variants functionally responsible for the HDL-C association, we performed fine-mapping analyses and selected 13 candidate variants that overlap putative regulatory regions to test for allelic differences in regulatory function. Of these variants, rs12463177-G increased transcriptional activity (1.5-fold, *P* = 0.004) and showed differential protein binding. Six additional variants (rs17699089, rs200788077, rs56322906, rs3760782, rs737337, and rs3745683) showed evidence of allelic differences in transcriptional activity and/or protein binding. Taken together, these data suggest a regulatory mechanism at the *ANGPTL8* HDL-C GWAS locus involving tissue-selective expression and at least one functional variant.

To date, at least 157 loci have been associated with high-density lipoprotein cholesterol (HDL-C) in genome-wide association studies (GWAS) (Willer *et al.* 2013). Analyses of European, African American, Mexican, Pima Indian, and East Asian participants have identified four different lead variants associated with HDL-C located in or near angiopoietin-like protein 8 (*ANGPTL8*) (Coram *et al.* 2013; Weissglas-Volkov *et al.* 2013; Willer *et al.* 2013; Nair *et al.* 2016; Spracklen *et al.* 2017). *ANGPTL8* is a small protein of 198 amino acids; the gene is located on chromosome 19 within intron 14 of dedicator of cytokinesis 6 (*DOCK6*), and is transcribed in the opposite direction of *DOCK6*. European GWAS meta-analyses identified rs737337 (*P* = 4.6 × 10^−17^, *N* = 185,000) (Teslovich *et al.* 2010; Willer *et al.* 2013) as the lead variant associated with HDL-C; this variant was also associated with total cholesterol (*P* = 4.1 × 10^−5^) but not triglycerides (*P =* 0.12) or low-density lipoprotein cholesterol (LDL-C, *P =* 0.26). rs737337 is located 2.8 kb upstream of the *ANGPTL8* transcription start site (RefSeq NM_018687) and is a synonymous variant in exon 19 of *DOCK6* (Thr723, RefSeq NM_020812). In an African American population, the lead variant rs12979813 was only associated with HDL-C (*P* = 1.9 × 10^−9^, *N* = 12,157) (Coram *et al.* 2013), which is located 7.5 kb upstream of *ANGPTL8* and in intron 22 of *DOCK6*. In a joint analysis of Mexican and Pima Indian samples, the lead variant rs2278426 was associated with HDL-C (*P* = 3.4 × 10^−9^, *N* = 4361) and total cholesterol (*P =* 5.0 × 10^−6^) (Weissglas-Volkov *et al.* 2013; Hanson *et al.* 2016), which is a nonsynonymous variant in exon 2 of *ANGPTL8* (Arg59Trp) that exhibits relatively high linkage disequilibrium (LD) with the European lead rs737337 [*r*^2^ = 0.74, 1000G Admixed American (AMR)] and moderate LD with the African American lead rs12979813 (*r*^2^ = 0.52 AMR). Finally, in recent lipid associations in East Asian samples, lead variant rs3760782 was the strongest HDL-C–associated variant (*P =* 8.8 × 10^−11^) and another variant in strong LD (rs1865063, *r*^2^ *=* 0.95) is the lead variant for total cholesterol (*P =* 1.5 × 10^−15^) and LDL-C (*P =* 1.8 × 10^−8^) (Spracklen *et al.* 2017). rs3760782 is located 3.5 kb upstream of *ANGPTL8* and in intron 20 of *DOCK6*. Given the extensive sharing of GWAS loci across populations (Visscher *et al.* 2012), we hypothesized that at least one shared variant at the *ANGPTL8* locus affects HDL-C in all of these populations.

*ANGPTL8* is a recently defined gene also called *C19orf80*, *LOC55908*, refeeding-induced fat and liver (*RIFL*), *TD26*, hepatocellular carcinoma-associated gene, lipasin, and betatrophin (Quagliarini *et al.* 2012; Ren *et al.* 2012; Zhang 2012). Serum ANGPTL8 protein levels have been associated with many metabolic phenotypes including type 1 and type 2 diabetes (Espes *et al.* 2014a,b; Fu *et al.* 2014b; Hu *et al.* 2014; Yamada *et al.* 2015), obesity (Fu *et al.* 2014b; Lee *et al.* 2016), and nonalcoholic fatty liver disease (Lee *et al.* 2016). The gene is mainly expressed in liver and adipose tissues, despite being entirely contained within one intron of the ubiquitously expressed *DOCK6* (Quagliarini *et al.* 2012; GTEx Consortium 2013). The precise mechanisms of action of ANGPTL8 remain unclear. ANGPTL8 is secreted into the plasma and is involved in triglyceride storage in adipose tissue; knockout mice gained less fat than wild-type (Wang *et al.* 2013) mice and *Angptl8* knockdown in 3T3L1 mouse adipocytes led to decreased triglyceride content (Ren *et al.* 2012). *ANGPTL8* expression is increased in response to stress, it upregulates early growth response transcription factor and downregulates adipose triglyceride lipase, suggesting a role in lipid homeostasis (Zhang *et al.* 2015). ANGPTL8 contains a lipoprotein lipase inhibitory motif and inhibits LPL function when coexpressed with ANGPTL3 (Haller *et al.* 2017). Additionally, serum ANGPTL8 protein is inversely associated with HDL-C levels (Yi *et al.* 2015) supporting a role of *ANGPTL8* in HDL-C metabolism.

*DOCK6* is a member of the dedicator of cytokinesis family of atypical guanine nucleotide exchange factors. *DOCK6* is expressed in many tissues, with highest levels of expression in the lungs, thyroid, and adipose (GTEx Consortium 2013). DOCK6 functions as a guanine nucleotide exchange factor for RAC1 and CDC42 and has roles in cytoskeletal remodeling and neurite outgrowth (Miyamoto *et al.* 2007). Mutations in *DOCK6* can cause Adams-Oliver syndrome, an actin cytoskeletopathy characterized by limb and skin defects (Shaheen *et al.* 2011). *DOCK6* does not have functions that obviously relate to HDL-C metabolism.

Two coding variants in ANGPTL8 have been proposed to affect HDL-C levels. A rare *ANGPTL8* nonsense variant, rs145464906 (MAF = 0.001 in >42,000 individuals of European ancestry) encoding Gln121Ter, is associated with increased HDL-C (*P* = 5.1 × 10^−13^) and increased triglycerides (*P =* 0.003) in an exome array-based association analysis (Peloso *et al.* 2014). Based on conditional analysis (Peloso *et al.* 2014) and LD (*r*^2^ < 0.01), rs145464906 is independent of the reported *ANGPTL8* GWAS variants. The common Mexican and Pima Indian lead variant, rs2278426 encoding Arg59Trp, has been proposed to increase cleavage of ANGPTL3, leading to decreased LPL activity and lower HDL-C (Hanson *et al.* 2016). Although coding variants have been identified, the variants responsible for the common HDL-C association signal at this locus remain unclear, and may include regulatory variants. While a simple mechanism involving a single variant that alters transcription of a single gene is straightforward, multiple variants and/or multiple genes may contribute to the functional consequences of a GWAS signal (Corradin *et al.* 2014; He *et al.* 2015; Roman *et al.* 2015).

In this study, we describe an association between the HDL-C locus variants and subcutaneous adipose level of *ANGPTL8* RNA. We show that a subset of HDL-C–associated variants overlap regions with strong evidence of regulatory activity (Bernstein *et al.* 2010; Encode Project Consortium 2012), and we use *trans*-ancestry fine-mapping and functional assays to identify variants exhibiting allelic differences in regulatory activity at the *ANGPTL8* HDL-C GWAS locus.

## Materials and Methods

### Study population and phenotypes

The METabolic Syndrome In Men (METSIM) study includes 10,197 men, aged from 45 to 73 yr, randomly selected from Kuopio, Eastern Finland, and examined in 2005–2010 (Stancakova *et al.* 2009; Laakso *et al.* 2017). The Ethics Committee of the University of Eastern Finland in Kuopio and the Kuopio University Hospital approved the METSIM study, and it was carried out in accordance with the Helsinki Declaration. Triglyceride and lipoprotein characteristics were measured via proton nuclear magnetic-resonance (NMR) or enzymatic assays in 10,079 METSIM participants (Inouye *et al.* 2010). DNA samples were genotyped on the Illumina OmniExpress and HumanCoreExome arrays, and additional genotypes were imputed using the GoT2D integrated haplotypes reference panel as previously described (Fuchsberger *et al.* 2016).

We also analyzed a set of 8421 self-identified African American participants from the Women’s Health Initiative SNP Health Association Resource (WHI-SHARe) study. Details of the study design, cohort characteristics, written informed consent, and study approval by local Human Subjects Committees have been described previously (Hays *et al.* 2003). Participants who had consented to genetic research were genotyped on the Affymetrix 6.0 array and additional genotypes were imputed using the 1000 Genomes Project data as a reference panel, as previously described (Reiner *et al.* 2012).

### Association, conditional, and haplotype analysis

For METSIM, we performed a preliminary test for association between ∼19 million genetic variants and 72 lipid and lipoprotein subclasses (Davis J.P., Huyghe J.R., Jackson A.U., Stringham H.M., Teslovich, T.M., Welch R.P., Fuchsberger C., Locke A.E., Narisu N., Chines P.S., Kangas A.J., Soininen P., Ala-Korpela M., Kuusisto J., Collins F.S., Laakso M., Mohlke K.L., Boehnke M., unpublished data). To better observe the relative differences between variants, we use the association of variants with the concentration of phospholipids in medium HDL because the association with this trait is stronger (*P* = 1.9 × 10^−7^) than with HDL-C (*P* = 7.7 × 10^−4^). We assumed an additive mode of inheritance and accounted for cryptic relatedness between individuals using the EMMAX mixed model (q.emmax test) implemented in EPACTS (Kang *et al.* 2010). After accounting for age, BMI, smoking status, and lipid-lowering medication status as covariates, trait value residuals were inverse normal transformed. We carried out reciprocal conditional analyses with candidate variants within the associated signal to evaluate the potential presence of a second association signal by adjusting for specific genetic variants, including METSIM lead variant rs737337 and Women’s Health Initiative (WHI) lead variant rs4804154. LD in locus plots was calculated from the METSIM imputed genotypes, and all chromosome coordinates correspond to hg19.

For WHI, association analysis was performed under an additive genetic model using linear regression adjusted for covariates. The imputed allelic dosage at each variant was tested via MACH2QTL (Li *et al.* 2010). Genome-wide African ancestry proportion, age, BMI, and smoking history were included as covariates. Genome-wide African ancestry proportion was derived from locus-specific ancestry, which has a correlation of 0.99 with the first principal component of population structure. Ancestry estimation has been described previously (Coram *et al.* 2013). To assess the potential presence of multiple, independent variants at the same locus influencing HDL-C trait, we repeated regression analyses, conditioning on rs4804154. LD in locus plots was calculated from WHI-imputed genotypes, and all chromosome coordinates correspond to hg19.

We constructed haplotypes based on five variants at *ANGPTL8* that were previously reported as the GWAS index variants (rs4804154, rs737337, and rs2278426) in studies of different ancestry groups (Teslovich *et al.* 2010; Coram *et al.* 2013; Weissglas-Volkov *et al.* 2013; Willer *et al.* 2013; Hanson *et al.* 2016) or are proxies (rs3745683 and rs3760782) in high LD with both the European and African American lead variants. We performed haplotype analyses using the “haplo.stat” R package (Schaid *et al.* 2002), estimated haplotypes and haplotype frequencies using the haplo.em function, and tested for association between haplotypes and the concentration of phospholipids in medium HDL (METSIM) or HDL-C (WHI) level using the haplo.glm function. We assumed an additive model in which the regression coefficient represented the expected change in inverse normalized HDL-C level with each additional copy of the specific haplotype compared with the reference haplotype. The same covariates used for single variant analysis were also used in the haplotype analysis model.

### Expression quantitative trait association

RNA from subcutaneous adipose tissue was extracted and the expression levels of probesets were measured using Affymetrix Human Genome U219 Array in 770 METSIM participants (Civelek *et al.* 2016). The expression quantitative trait locus (eQTL) for *ANGPTL8* was not previously reported because of a gene name difference with RefSeq and Ensembl. Expression data were normalized using Robust Multi-Array Average (RMA) analysis (Irizarry *et al.* 2003). eQTL associations were performed as previously described (Civelek *et al.* 2016). Briefly, we applied PEER analysis (Stegle *et al.* 2010) to account for complex nongenetic factors in the RMA-normalized gene expression levels. We adjusted for 35 inferred confounding factors and inverse normal transformed the PEER-processed residuals. In eQTL analysis, we used genotype dosages imputed using Haplotype Reference Consortium data (McCarthy *et al.* 2016) to test for the variant association with expression levels of all genes within 1 Mb of rs737337. We assumed an additive mode of inheritance and accounted for cryptic relatedness between individuals using the EMMAX mixed model implemented in EPACTS (Kang *et al.* 2010). Additionally, we performed conditional analysis on the expression level of *ANGPTL8* by adjusting for rs4804155 and on the expression level of *DOCK6* by adjusting for rs17699089 to assess the presence of multiple, independent associations. We also performed conditional analysis using candidate functional variant rs12463177 to assess the coincidence of GWAS and eQTL signals. To examine the relationship between RMA-normalized expression levels and HDL-C, we adjusted both traits for age and BMI, inverse normal transformed the residuals, and then tested for association in regression analyses.

### MANTRA

To fine-map the *ANGPTL8* locus, we performed *trans*-ancestry meta-analysis using MANTRA (Morris 2011). MANTRA accounts for the shared similarity in closely related populations using Bayesian partition model assuming the same underlying allelic effect. It models the effect heterogeneity among distant populations by clustering according to the shared ancestry and allelic effects. We conducted the analysis based on the association summary statistics from GWAS in METSIM and WHI and included variants present in both METSIM and WHI. We constructed a 99% credible set of variants by ranking all variants according to their Bayes factors.

### CAVIAR and PAINTOR

To prioritize functional variants, we analyzed variants at the *ANGPTL8* locus using both CAVIAR (Hormozdiari *et al.* 2014) and PAINTOR (Kichaev *et al.* 2014). CAVIAR estimates the posterior probability of a variant being functional by jointly modeling the *P*-values and β association statistics, and PAINTOR leverages functional genomic annotation data, in addition to association strength, to prioritize functional variants. Both methods allow for multiple functional variants at the risk locus. Using consistent alleles for METSIM and WHI African Americans, we calculated *Z* scores based on *P*-values and the sign of β from GWAS in these studies. For METSIM, the LD matrix was calculated based on European data from the 1000 Genomes Project Phase 1. For WHI African Americans, LD was calculated based on imputed data as previously described (Reiner *et al.* 2012). In CAVIAR, we returned the credible set that contains all of the true functional variants with 95% confidence level. The annotation matrix used in PAINTOR contained data from the ENCODE project accessed from the UCSC Genome Browser using the Table Browser tool. The matrix included HepG2 H3K4me1, H3K4me2, H3K4me3, and H3K27ac histone marks, DNase hypersensitivity clusters (ENCODE v3) and transcription factor binding (ENCODE v2) from the Broad Institute. Presence or absence of overlap was determined by the UCSC Table Browser intersection with the signal tracks. In PAINTOR, analysis was performed for METSIM alone (Finns), WHI alone (African Americans), and the two data sets together. We set the number of functional variants as 2, 3, 4, or 5 based on feasible running time. PAINTOR predicted variants to be functional based on a posterior probability >0.1, a threshold suggested previously (Kichaev *et al.* 2014).

### Functional annotation

To identify regulatory overlap of HDL-C–associated variants with histone marks, transcription factor binding, and DNase hypersensitivity sites, we used data from the ENCODE and Roadmap Epigenome projects accessed through the UCSC genome browser (Bernstein *et al.* 2010; Encode Project Consortium 2012). RNA-seq data were obtained from the Roadmap Epigenome Project (Bernstein *et al.* 2010; adult liver and adipose tissue) or a previous publication (Parker *et al.* 2013; HepG2). We evaluated previously published positive correlations of DNase hypersensitivity sites with gene expression in liver cell types (Sheffield *et al.* 2013). LD calculations for selecting candidate variants were based on 1000 Genomes Phase 1 data. The AFR dataset was used for African American LD and EUR for European LD.

### Cell culture

HepG2, SW872, SGBS, 293T, 3T3L1, Huh-7, and MIN6 cell lines were maintained at 37° with 5% CO_2._ HepG2 cells (ATCC, HB-8065) were cultured in MEM-α (Gibco) supplemented with 10% FBS and 1 mM sodium pyruvate. SW872 cells (ATCC, HTB92) were cultured in DMEM:F12 (Sigma) supplemented with 10% FBS. SGBS cells (Wabitsch *et al.* 2001) were generously provided by Dr. Martin Wabitsch (University of Ulm) and cultured in DMEM:F12 (Sigma) supplemented with 10% FBS and 5% 3.3 mM biotin/1.7 mM panthotenate solution. SW872 and SGBS cells were transfected in the undifferentiated, preadipocyte state. 293T cells (ATCC, CRL-3216) were cultured in DMEM (Sigma) supplemented with 10% FBS and 200 mM L-glutamine. 3T3-L1 cells (ATCC, CL-173) were cultured and differentiated as described in the ATCC protocol. Huh-7 cells (JCRB0403, Japanese Collection of Research Bioresources Cell Bank, National Institute of Biomedical Innovation), were cultured in DMEM with high glucose (Gibco) with 10% FBS, 1 mM sodium pyruvate, 1 mM nonessential amino acids (Sigma), and 1 mM L-glutamine. MIN6 cells (Miyazaki *et al.* 1990) were cultured in DMEM (Sigma), supplemented with 10% FBS, 1 mM sodium pyruvate, and 0.1 mM β-mercaptoethanol.

### Dual luciferase transcriptional reporter assays

We PCR-amplified fragments surrounding each regulatory region or variant with five PRIME Mastermix (five PRIME) or Phusion High-Fidelity Polymerase (New England Biosystems) with the primers listed in Supplemental Material, Table S1 in File S1 from DNA of individuals homozygous for alleles associated with increased or decreased HDL-C. Gateway attB sites were included in primers and Gateway cloning was used to insert fragments into a Gateway-compatible pGL4.23 (minimal promoter) or pGL4.10 (promoterless) firefly luciferase reporter vector (Promega). Fragments containing HDL-C–associated variants are designated as forward or reverse based on their orientation with respect to the genome and the *ANGPTL8* gene. The 5-kb and promoter regions were only cloned in the forward orientation (pGL4.10) because they include the *ANGPTL8* promoter. Regions were isolated based on epigenetic marks of promoter and/or enhancer regions surrounding the HDL-C–associated variants. We isolated three to five independent clones (biological replicates) for each allele for each orientation and verified by sequencing. Each clone was cotransfected with *Renilla* luciferase vector in duplicate (HepG2, 293T, Huh-7, SW872) or triplicate (SGBS, 3T3L1) wells (technical replicates) using FUGENE 6 (HepG2, 293T, Huh-7, SW872, 3T3L1; Promega), Lipofectamine 2000 (Min6; Life Technologies), or Lipofectamine 3000 (SGBS; Life Technologies). Twenty-four (SGBS) or 48 h (all other cell types) after transfection, we collected cell lysates and assayed for luciferase activity using the Dual-Luciferase Reporter Assay System (Promega). Firefly luciferase activity of the clones containing the PCR fragments was normalized to *Renilla* luciferase readings to control for differences in transfection efficiency. We repeated all luciferase transcriptional reporter experiments on independent days and obtained consistent results. Data are reported as fold change in activity relative to an empty pGL4.23 or pGL4.10 vector. We used two-sided Student’s *t*-tests to compare luciferase activity between alleles or haplotypes.

### Electrophoretic mobility shift assays

For electrophoretic mobility shift assays (EMSAs), we prepared nuclear cell extracts from HepG2, HuH-7, SGBS, 3T3L1, SW872, and MIN6 cells using the NE-PER nuclear and cytoplasmic extraction kit (Thermo Scientific). Protein concentration was measured with a BCA assay (Thermo Scientific), and lysates were stored at −80° until use. We designed 17–19 bp biotin– or IR-Dye 700–labeled oligos around the HDL-C–associated variants (Table S1 in File S1) for both alleles. We annealed double-stranded oligos and performed binding reactions as previously described (Kulzer *et al.* 2014). We used 4–6 μg of antibody in supershift assays and 100–200 ng CEBPB-purified protein for select EMSAs. Binding reactions were run on nondenaturing PAGE DNA retardation gels in 0.5× TBE (Lonza). For biotin-labeled oligos, we transferred the reactions to Biodyne nylon membranes (Thermo Scientific), cross-linked them on a UV cross-linker (Stratagene), and detected DNA–protein complexes by chemiluminescence. For IR-DYE 700–labeled oligos, we imaged gels on a LiCor Odyssey CLx Imaging System. We repeated all EMSA experiments on another day with consistent results.

### Data availability

Plasmids are available upon request. Supplemental figures and tables contain all relevant data.

## Results

### Regulation of tissue-selective expression of ANGPTL8

**Figure 1:**
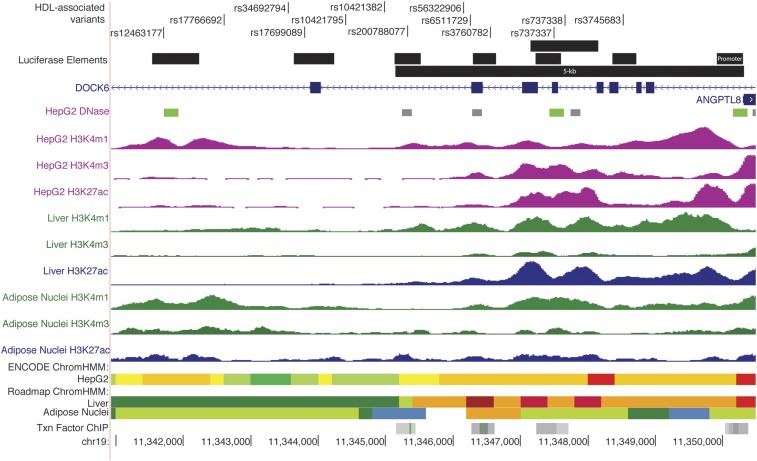
Thirteen variants overlap predicted regulatory regions. Thirteen variants overlap regulatory regions defined by histone marks, chromatin accessibility, and transcription factor binding. The full set of 42 candidate variants span a 39-kb region (Figure S4 in File S1). Green rectangles denote DNase hypersensitivity peaks correlated with *ANGPTL8* expression across 112 cell lines in a previous study (Sheffield *et al.* 2013). DNase hypersensitivity correlation with *DOCK6* expression is not indicated because the correlated peaks do not overlap HepG2 or hepatocyte DNase peaks. Gray rectangles represent transcription factor binding; the identities of transcription factors are listed in Table S5 in File S1. Data were accessed from ENCODE, the Epigenome Roadmap Atlas, and the UCSC Genome Browser. ENCODE ChromHMM: gray, heterochromatin; blue, insulator; green, transcription; yellow and orange, enhancer; red and pink, promoter. Roadmap ChromHMM: orange, enhancer; light green, genic enhancer; dark green, transcription; blue, heterochromatin; red, promoter. Black rectangles represent regions analyzed in transcriptional reporter assays.

**Figure 2:**
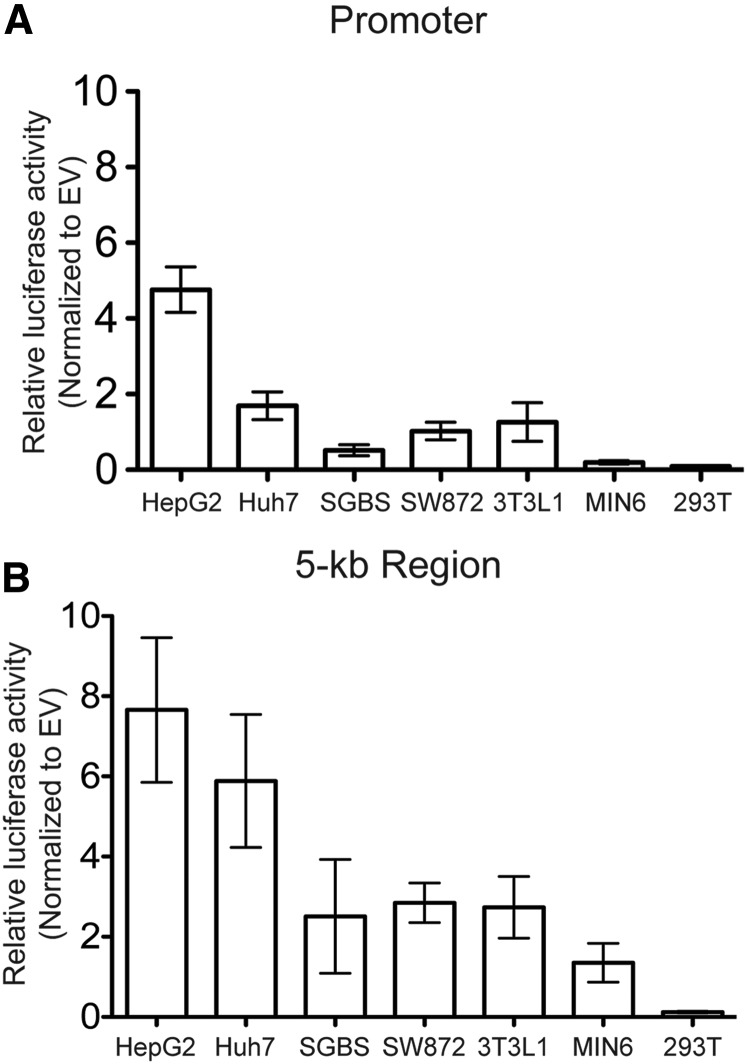
Cell-type specificity of *ANGPTL8* is influenced by nearby regulatory regions. Candidate regulatory regions were tested in a pGL4.10 vector in six cell types to determine drivers of tissue specificity. Reporter activity was normalized to empty vector (EV) in each cell type. Data are represented as the mean ± SD of 10 biological replicates. (A) A 400-bp promoter. (B) A 5-kb regulatory region including the promoter. Comparison of the 400-bp promoter *vs.* 5-kb regulatory region; *P* < 0.05 for cell types. Cell lines: HepG2, human hepatocellular carcinoma (liver); Huh-7, human hepatocellular carcinoma; SGBS, human preadipocyte; SW872, human adipocyte; 3T3L1, mouse adipocyte; MIN6, mouse pancreatic β cell; 293T, human embryonic kidney.

*ANGPTL8* expression is largely restricted to liver and adipose tissues (Quagliarini *et al.* 2012; GTEx Consortium 2013). To determine drivers of *ANGPTL8* tissue specificity, we tested candidate regulatory regions in human hepatocyte (HepG2, Huh-7), human preadipocyte (SGBS), human adipocyte (SW872), mouse adipocyte (3T3-L1), human embryonic kidney (293T), and mouse islet β (MIN6) cell lines. We tested a region extending 400 bp upstream of the transcription start site that spans epigenetic marks characteristic of promoters, and a regulatory region that extends 5 kb upstream of the transcription start site (regions shown in Figure 1). The 400 bp promoter contains no HDL-C–associated variants; the 5-kb region contains seven HDL-C–associated variants (rs200788077, rs56322906, rs6511729, rs3760782, rs737337, rs737338, and rs3745683). We tested these candidate regions in transcriptional reporter luciferase assays. In comparison to empty vector, the promoter increased transcriptional activity fivefold in HepG2 (*P* < 0.0001) and 1.6-fold in Huh-7 (*P* = 0.04), but not in any other cell type (*P >* 0.5, Figure 2A). The 5-kb region increased transcriptional activity in HepG2 by eightfold in HepG2 (*P =* 0.0007) and sixfold in Huh-7 (*P =* 0.002) compared with empty vector and increased transcriptional activity twofold in preadipocyte (SW872, *P =* 0.0007) and adipocyte (3T3-L1, *P* = 0.023) cells compared with empty vector (Figure 2B). Neither region showed transcriptional activity in MIN6 or 293T cells. These data suggest that the 400-bp region contains promoter regulatory elements contributing to tissue specificity in liver, but may be mediated by additional enhancer elements, especially in adipocytes.

### Characterization of the ANGPTL8 HDL-C GWAS locus

**Figure 3:**
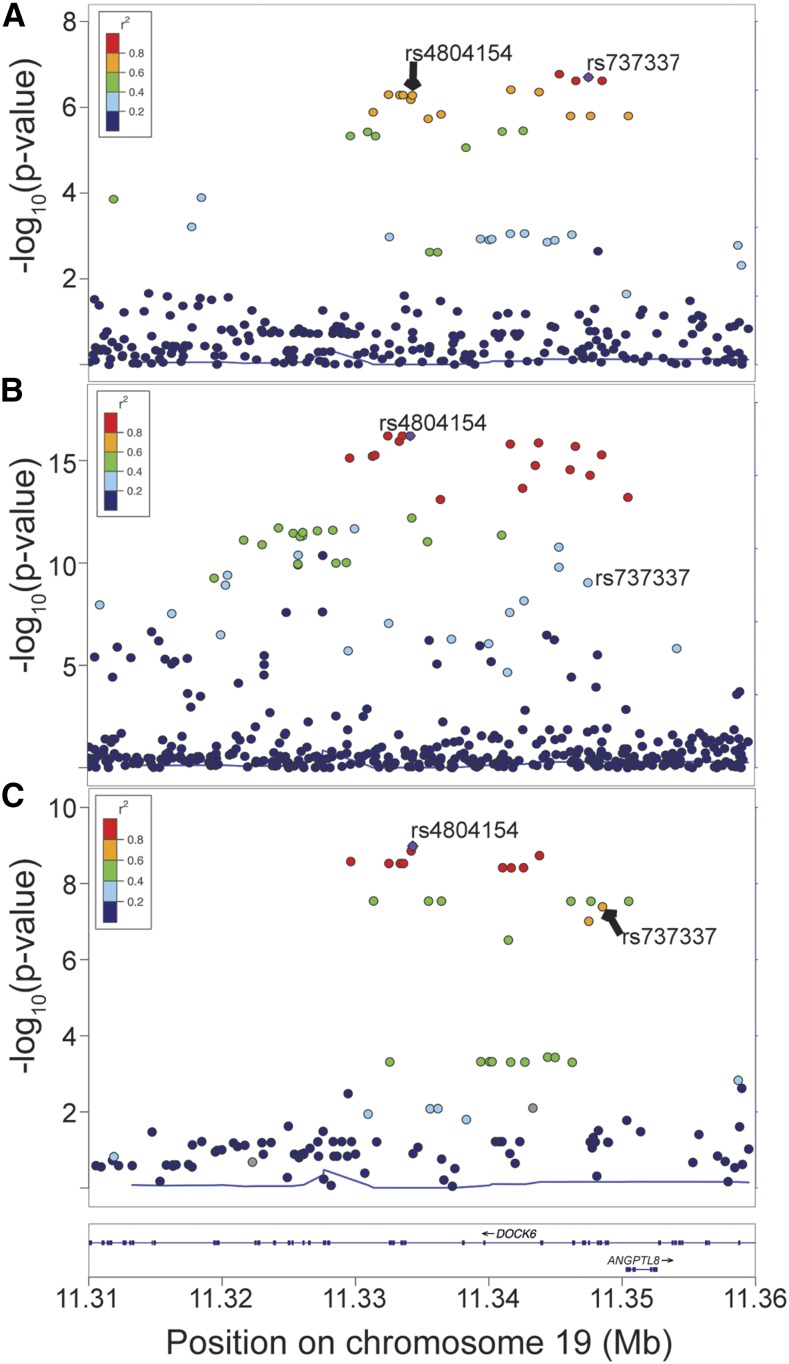
Locus plots of HDL-C and eQTL associations near *ANGPTL8*. (A) Concentration of phospholipids in medium HDL in the METSIM study of Finnish individuals (*N* = 8380). Variants are colored according to LD (*r*^2^) with rs737337 (purple), the lead variant in European meta-analyses by GLGC. (B) HDL-C association in the WHI subset of African American individuals (*N* = 8244). Variants are colored according to LD (*r*^2^) with rs4804154 (purple). (C) HDL-C–associated variants are also associated with *ANGPTL8* expression in 770 subcutaneous adipose samples from the METSIM study. The European and African American lead variants (rs737337 and rs4804154, labeled in plots) are among the most significant variants. Variants are colored according to LD (*r*^2^) with rs4804154 (purple).

To characterize and determine shared variants of the *ANGPTL8* association signal across populations, we analyzed the METSIM study of Finns (*N* = 8380) and the African Americans subset (*N* = 8244) from the WHI (Coram *et al.* 2013). In a preliminary METSIM analysis, the variants most strongly associated with HDL-C at the *ANGPTL8* locus were rs737337 (*P* = 7.7 × 10^−4^, β = −0.09) and its LD proxies; these variants were more strongly associated with the concentration of phospholipids in medium HDL particles (*P* = 1.9 × 10^−7^, β = −0.14; Figure 3A and Table S2 in File S1) than HDL-C (*P* = 7.7 × 10^−4^, β = −0.14) (Davis J.P., Huyghe J.R., Jackson A.U., Stringham H.M., Teslovich, T.M., Welch R.P., Fuchsberger C., Locke A.E., Narisu N., Chines P.S., Kangas A.J., Soininen P., Ala-Korpela M., Kuusisto J., Collins F.S., Laakso M., Mohlke K.L., Boehnke M., unpublished data). Association analyses conditioned on rs737337 showed no evidence for any additional signals (all *P* > 8.0 × 10^−4^, Figure S1B and Table S2 in File S1). In WHI, the lead variant associated with HDL-C is rs4804154 (*P* = 8.4 × 10^−17^), located 13 kb from rs737337, and we did not observe evidence of any additional signals (*P* > 4.8 × 10^−4^, Figure 3B, Figure S1D, and Table S3 in File S1). rs4804154 is in low LD with the reported African American lead variant rs12979813 (*r*^2^ = 0.18 AFR). rs12979813 is the most strongly associated genotyped variant in the previously reported African American sample (Coram *et al.* 2013), which includes the WHI study, but rs4804154 is the strongest variant after imputation in WHI. These two lead variants, rs737337 and rs4804154, are in moderate pairwise LD in Europeans (*r*^2^ = 0.67 EUR) and in low pairwise LD in Africans (*r*^2^ = 0.26 AFR). rs737337 was among the most strongly associated variants in WHI (*P* = 9.1 × 10^−10^) and the same was true of rs4804154 in METSIM (*P* = 5.7 × 10^−5^). The rare coding variant rs145464906 in *ANGPTL8* (Peloso *et al.* 2014) is not present in METSIM and was not significantly associated with HDL-C in WHI (*P* = 0.30). Conditioning on the common coding variant rs2278426 attenuates, but does not abolish, the signal in both populations (METSIM rs737337, *P* = 0.03; WHI rs4804154, *P =* 1.82 × 10^−4^). Conditioning on the lead variant abolished the association with rs2278426 (WHI, *P =* 0.78; METSIM, *P =* 0.86), suggesting that the regulatory variants capture more of the association signal than rs2278426 alone. Because many GWAS loci are shared across populations, the presence of an association signal in both WHI and METSIM supports the hypothesis of at least one shared functional variant across populations.

To further characterize the locus across populations, we conducted haplotype association analyses in METSIM and WHI (Table 1). By comparing variants across haplotypes and populations, we can hypothesize which variants represent the inheritance pattern of variants that have a functional effect. We included five variants in the analyses: the lead variants from European (rs737337), African American (rs4804154), Mexican/Pima Indian (rs2278426), and East Asian (rs3760782) studies and the one variant (rs3745683) in high LD (*r*^2^ > 0.8) with the leads in all four studies. The most common haplotype in both METSIM and WHI contained alleles individually associated with increased HDL-C (haplotype 1, Table 1). In both studies, the haplotype containing all the alleles individually associated with decreased HDL-C (haplotype 3) showed the strongest association with HDL-C ($\hat{\text{β}}$ = −0.044, *P <* 1.0 × 10^−22^ in WHI and $\hat{\text{β}}$ = −0.146, *P* = 5.3 × 10^−7^ in METSIM). Haplotypes 1 and 2 differed only for rs737337 alleles. Haplotype 2 is common in African Americans (21% frequency) and nearly absent in Finns (0.02% frequency), which explains the different extent of *r*^2^-based LD in these populations. The small effect sizes of haplotype 2 ($\hat{\text{β}}$= −0.01 in WHI), 3 ($\hat{\text{β}}$ = −0.044 in WHI, $\hat{\text{β}}$ = −0.146 in METSIM), and 5 ($\hat{\text{β}}$ = −0.009 in WHI, $\hat{\text{β}}$ = −0.049 in METSIM) suggest that rs737337, rs2278426, and rs4804154 may contribute to, but are not alone responsible for, the association signal. These data are consistent with a signal shared across populations driven by one or more functional variants represented by rs3745683 and rs3760782, with potential additional contributions from rs737337, rs2278426, and/or other proxies.

**Table 1:** Haplotype association analyses in the WHI and METSIM studies

							Haplotype 1 Reference			Haplotype 3 Reference			Haplotype 4 Reference		
	rs4804154	rs3760782	rs737337	rs3745683	rs2278426	Freq.	Effect	SE	*P* Value	Effect	SE	*P* Value	Effect	SE	*P* Value
WHI African American Participants															
1	C	C	T	G	C	0.59	REF	REF	REF	0.042	0.005	8.8E-16	0.019	0.016	0.225
2	C	C	**C**	G	C	0.21	−0.010	0.055	0.051	0.032	0.006	1.5E−07	0.009	0.016	0.564
3	**T**	**T**	**C**	**A**	**T**	0.18	−0.044	0.005	<1E−22	REF	REF	REF	−0.025	0.016	0.126
4	**T**	**T**	**C**	**A**	C	0.01	−0.035	0.019	0.069	0.006	0.020	0.760	REF	REF	REF
5	**T**	C	T	G	C	0.01	−0.009	0.026	0.735	0.040	0.026	0.125	0.014	0.031	0.661
METSIM Finnish participants															
1	C	C	T	G	C	0.88	REF	REF	REF	0.147	0.029	4.4E−07	0.124	0.056	0.029
2	C	C	**C**	G	C	0.0002	N/A	N/A	N/A	N/A	N/A	N/A	N/A	N/A	N/A
3	**T**	**T**	**C**	**A**	**T**	0.06	−0.146	0.029	5.3E−07	REF	REF	REF	−0.023	0.063	0.720
4	**T**	**T**	**C**	**A**	C	0.02	−0.120	0.056	0.034	0.027	0.063	0.660	REF	REF	REF
5	**T**	C	T	G	C	0.03	−0.049	0.040	0.210	0.097	0.048	0.041	0.074	0.068	0.280

Haplotype association analyses in 8244 African Americans in WHI and 8380 Europeans in METSIM. Alleles shown in bold differ from haplotype 1. In both studies, alleles shown in haplotype 3 were individually associated with decreased HDL-C. Freq., haplotype frequency; REF, reference haplotype for interpreting association analyses; N/A, haplotype was too rare to be analyzed.

### eQTL associations with HDL-C–associated variants and nearby genes

To determine which gene(s) the HDL-C–associated variants may affect, we investigated eQTL associations. We observed an eQTL association in subcutaneous adipose tissue from 770 METSIM study participants for both *ANGPTL8* (rs4804154, *P* = 1.0 × 10^−9^; Figure 3C, Figure S2 in File S1, and Table 2) and *DOCK6* (rs17699089, *P* = 7.2 × 10^−7^; Figure S2 in File S1, Table 2, and Table S4 in File S1), but not any other gene within 1 Mb of rs737337 (Table S4 in File S1). The METSIM and WHI HDL-C–associated variants were among the variants most strongly associated with both *ANGPTL8* and *DOCK6* mRNA levels (Figure 3 and Table S4 in File S1), suggesting that the same variants associated with HDL-C may act by affecting expression level of *ANGPTL8* and/or *DOCK6*. Conditional analyses were performed to confirm the coincidence of the GWAS and eQTL signals. For both genes, alleles associated with lower HDL-C levels were associated with lower mRNA levels. In addition, *ANGPTL8* mRNA level was associated with HDL-C level in METSIM samples (*P* = 0.017, Figure S3 in File S1), whereas *DOCK6* was not (*P* = 0.42, Figure S3 in File S1). Evidence that the variants most strongly associated with HDL-C are also most strongly associated with *ANGPTL8* mRNA levels suggests that a regulatory mechanism acts at this GWAS locus.

**Table 2:** Associations with *ANGPTL8* and *DOCK6* expression in subcutaneous adipose tissue

Variant	Alleles*^a^*	Gene	Effect*^b^*	SE	*P*-Value	*P*_cond_-eSNP	*P*_cond_-rs12463177	*r*^2^ with eSNP (EUR)*^c^*	*r*^2^ with eSNP (AFR)*^c^*
rs4804155	G/C	*ANGPTL8*	−0.499	0.081	1.04 × 10^−9^	—	0.516	—	—
rs737337	C/T	*ANGPTL8*	−0.526	0.098	9.74 × 10^−8^	0.244	0.402	0.67	0.40
rs4804154	T/C	*ANGPTL8*	−0.500	0.081	1.38 × 10^−9^	0.762	0.270	1.00	0.48
rs12463177	C/G	*ANGPTL8*	−0.479	0.080	3.84 × 10^−9^	0.570	—	0.94	0.47
rs17699089	G/A	*DOCK6*	−0.406	0.081	7.21 × 10^−7^	—	0.005	—	—
rs737337	C/T	*DOCK6*	−0.298	0.099	2.68 × 10^−3^	0.324	0.413	0.74	0.29
rs4804154	T/C	*DOCK6*	−0.398	0.082	1.65 × 10^−6^	0.253	0.267	0.94	0.91
rs12463177	C/G	*DOCK6*	−0.382	0.081	3.06 × 10^−6^	0.029	—	1.00	0.98

Lead eQTL variants for *ANGPTL8* (rs4804155) and *DOCK6* (rs17699089), lead GWAS variants (rs4804154 and rs737337), and functional candidate variant rs12463177 association with *ANGPTL8* and *DOCK6* expression in 770 primary subcutaneous adipose samples. Conditional analysis on each lead eSNP and the candidate functional variant rs12463177 attenuated both the *ANGPTL8* and *DOCK6* association signals. *P*_cond_, conditional *P*-value; eSNP, strongest associated eQTL variant.

a The HDL-C–decreasing and eQTL effect alleles are presented first.

b Effect size is shown as the inverse normal transformed expression levels [log_2_(fluorescence intensity)] with each additional copy of the allele.

c *r*^2^ is calculated from 1000 Genomes Phase 1 data.

### Selection of variants to test for allelic differences in regulatory activity

Prioritizing variants at GWAS loci for functional study can be challenging, especially at loci with regulatory mechanisms. To narrow variants that may have regulatory function, we considered three methods: LD with the lead GWAS variants, three fine-mapping algorithms in each of METSIM and WHI, and overlap with predicted regulatory regions (Figure S4 in File S1). First, we considered variants in LD with the lead GWAS variants in European and/or African American ancestry individuals (1000 Genomes EUR and AFR). Because the variants in Europeans are of moderate allele frequency (MAF ∼0.07), we considered variants that meet an LD threshold *r*^2^ > 0.5. In total, 42 variants exhibited *r*^2^ > 0.5 with a lead HDL-C–associated variant in at least one study and could be considered as candidates for regulatory function (Figure S5 and Table S5 in File S1). As a second approach to prioritize candidate variants at this locus, we used the MANTRA, CAVIAR, and PAINTOR algorithms to interpret the HDL-C–associated variants in the METSIM Finnish and WHI African American ancestry groups (Table S6, Table S7, and Table S8 in File S1). Finally, we identified candidate variants based on simple positional overlap with evidence of predicted regulatory regions, as variants overlapping these regions are more likely to be functional (Figure S5 and Table S5 in File S1) (Bernstein *et al.* 2010; Encode Project Consortium 2012; Parker *et al.* 2013; Sheffield *et al.* 2013). Nine variants identified in at least one fine-mapping analysis overlapped regulatory regions (rs12463177, rs17766692, rs17699089, rs200788077, rs56322906, rs3760782, rs737337, rs737338, and rs3745683). We considered these nine variants to represent the most plausible candidate variants based both on fine-mapping and regulatory overlap and examined them for allelic differences on regulatory function. To compare the set of nine most plausible candidate variants to those that only show regulatory overlap, we also examined four additional variants in regulatory regions that were not identified by any fine-mapping analysis (rs34692794, rs10421795, rs10421382, and rs6511729). Thus, we examined a total of 13 variants in assays examining allelic effects on regulatory function. All 13 variants are located within 9 kb of the *ANGPTL8* transcription start site (Figure 1 and Table S5 in File S1).

### Functional characterization of candidate variants

**Figure 4:**
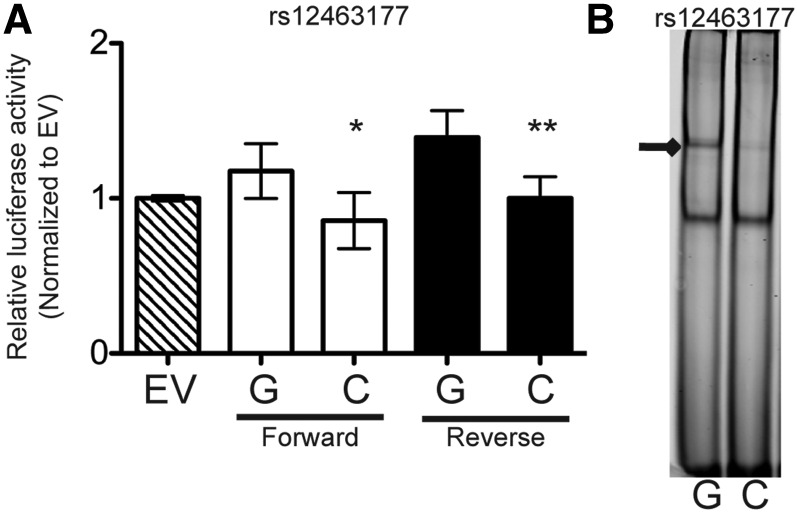
Allelic differences in regulatory assays of rs12463177 (A) A 698-bp region containing either allele of rs12463177 was cloned into the pGL4.23 vector and transfected in HepG2 cells. Data are represented as the mean ± SD of 3–5 biological replicates. Luciferase activity was normalized to empty vector (EV) and *P*-values were determined by *t*-tests. * *P* < 0.05, ** *P* < 0.01. (B) Differential protein binding was evaluated *in vitro* using EMSA. IR-labeled probes containing either allele of rs12463177 were incubated with 10 μg HepG2 nuclear protein. The arrow shows stronger binding for rs12463177-G. Consistent binding was observed with SGBS nuclear protein (Figure S7 in File S1). HDL-C–increasing alleles are presented first.

We examined the candidate variants for allelic differences in assays of regulatory function in human liver-derived (HepG2, Huh-7), preadipocyte (SGBS), and adipocyte (SW872) cells. We chose these cell types because *ANGPTL8* is expressed in liver and adipose, they had the highest transcriptional activity in our cell type-specificity assays, and they have roles in HDL-C metabolism. We tested variants individually or as a haplotype in luciferase transcriptional reporter assays and/or in EMSA (Figure S6 and Figure S7 in File S1). Among the variants analyzed, rs12463177, which was identified in all three fine-mapping analyses and is a candidate at *r*^2^ > 0.5 in both populations (*r*^2^ *=* 0.74 with rs737337 EUR, *r*^2^ *=* 0.93 with rs4804154 AFR), and is 8 kb upstream from the *ANGPLT8* transcription start site showed significant (*P* < 0.05) allelic differences in two assays of regulatory function (Figure 4). In luciferase transcriptional reporter assays in HepG2, a 697-bp region containing rs12463177-G showed 1.2 to 1.4-fold increased enhancer activity (*P* = 0.02 forward orientation; *P* = 0.004 reverse) compared with rs12463177-C. While modest, this allelic difference was replicated in three independent experiments. The significant effect on transcriptional activity was not replicated in Huh-7, SGBS, and SW872 cells, in which this region showed repressor activity; however, the trend between the alleles is consistent with HepG2, at least in the reverse orientation (Figure S6 in File S1). In EMSAs using HepG2 and SGBS nuclear extract, the rs12463177-G allele showed increased binding (Figure 4, and Figure S7 and Figure S8 in File S1). The direction of effect of rs12463177 transcriptional activity is consistent with the eQTL direction. A functional role for rs12463177 is consistent with additional evidence that rs12463177 overlaps a DNase hypersensitivity site that was previously correlated to *ANGPTL8* expression (Sheffield *et al.* 2013; green rectangle in Figure 1) and that the eQTL association signal is attenuated when conditioned on rs12463177 (Figure S2 in File S1 and Table 2).

**Figure 5:**
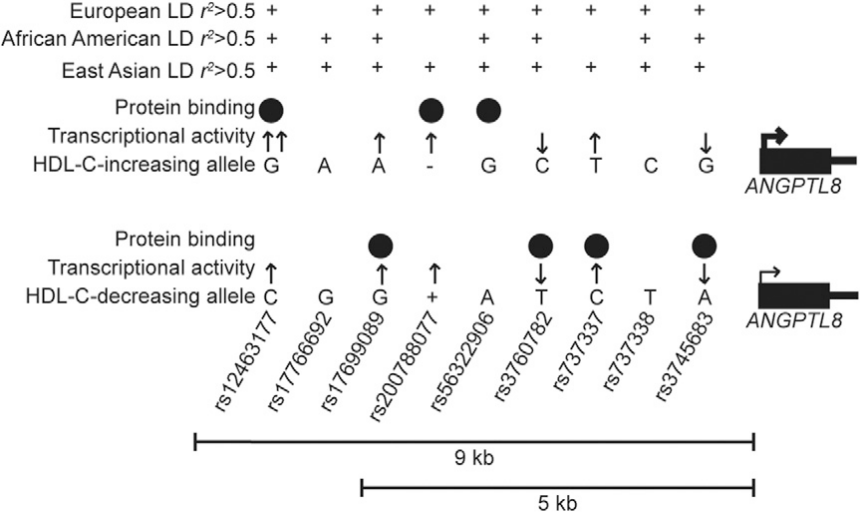
Summary of functional results for seven candidate variants. Summary of shared LD with lead HDL-C–associated variants reported in European (rs737337), African American (rs4804154), and East Asian (rs3760782) populations are shown; variants meeting the *r*^2^ > 0.5 threshold are marked with +. Results from transcriptional reporter luciferase assays and electrophoretic mobility shift assays (EMSA) for nine candidate variants are shown. Arrows show the direction of transcriptional activity in reporter assays. Two arrows at rs12463177-G indicate allelic differences in transcriptional activity. Black circles show allele-specific protein binding observed in EMSA experiments. The larger and smaller arrows at the *ANGPTL8* promoter indicate higher and lower expression level from adipose eQTL data. Variants are located within 9 kb of the *ANGPTL8* transcription start site; variant distances are not drawn to scale.

Six additional variants (rs17699089, rs200788077, rs56322906, rs3760782, rs737337, and rs3745683) showed evidence of allelic differences in an assay for regulatory function and two (rs17766692 and rs737338) did not (Figure S6, Figure S7, Figure S8, and Figure S9 in File S1). rs56322906 was only tested in transcriptional reporter assays as part of the 5-kb haplotype described below (Figure S11 in File S1). rs737337 showed by far the strongest enhancer activity in transcriptional reporter assays, an up to 60-fold increase compared with empty vector in HepG2 (Figure S6 in File S1), and rs737337-C shows strong allele-specific binding in liver cell types (Figure S7 in File S1). Supershift experiments using HepG2 nuclear extract identified RXRα as binding to this probe, although not as part of the allele-specific complex (Figure S10 in File S1). Interestingly, the four variants that overlap regulatory regions but were not predicted by any fine-mapping analysis (rs34692794, rs10421795, rs10421382, and rs6511729) did not show allelic differences in protein binding (Figure S9 in File S1). In total, we observed evidence of functional activity for seven of the nine candidate regulatory variants in these assays (Figure 5).

To examine the effect on transcriptional activity of multiple variants together with the *ANGPTL8* promoter, we tested 5-kb haplotypes located immediately upstream of the *ANGPTL8* transcription start site. This region includes five of the seven variants showing allelic differences in protein binding and/or transcriptional activity when examined separately (rs200788077, rs56322906, rs3760782, rs737337, and rs3745683; Figure S6 in File S1) and two variants that did not show evidence of allelic differences (rs6511729 and rs737338; Figure S9 in File S1). Transcriptional reporter assays of the smaller segments containing individual variants had shown both activator (*e.g.*, rs737337) and repressor (*e.g.*, rs3760782 and rs3745683) activity. The 5-kb haplotype acted as an enhancer in HepG2, Huh-7, SGBS, and SW872, with intermediate activity observed between the individual segments, but did not show significant differences in transcriptional activity between the two haplotypes (Figure S11 in File S1). These results suggest a complex regulatory mechanism involving enhancer and repressor regions that work in concert to regulate expression.

## Discussion

In this study, we examined the tissue specificity of *ANGPTL8*, reported the first eQTL for variants at the *ANGPTL8* HDL-C GWAS locus in adipose tissue, and identified variants at the GWAS locus that exhibit allelic differences in assays of regulatory function. We found that a 400-bp promoter is the main driver of tissue specificity in liver, and that expression may be mediated by additional enhancer elements within 5 kb upstream of *ANGPTL8*, especially in adipocytes. Of seven candidate regulatory variants that showed allelic differences in transcriptional activity and/or protein binding, rs12463177 showed the clearest allelic differences consistent with the direction of the *ANGPTL8* eQTL. These data suggest that multiple regions and potentially multiple variants regulate *ANGPTL8* expression in liver and adipose tissue.

*ANGPTL8* is a strong candidate gene at this GWAS locus. Although we observed coincidental association between the GWAS variants and transcript level for both *ANGPTL8* and *DOCK6* in subcutaneous adipose tissue samples, *ANGPTL8* mRNA level was associated with HDL-C in METSIM, whereas *DOCK6* mRNA level was not (Figure S3 in File S1). DNase hypersensitivity sites that overlap regulatory variants are correlated to *ANGPTL8* expression (Figure 1), providing further support for *ANGPTL8* as the target gene. *ANGPTL8* protein levels have been shown to be inversely associated with HDL-C (Yi *et al.* 2015). This direction is opposite of the association we observed with *ANGPTL8* expression and HDL-C; however, others have suggested that ELISA methods may not consistently quantify serum ANGPTL8 levels (Fu *et al.* 2014a). Furthermore, ANGPTL8 inhibits lipoprotein lipase when coexpressed with ANGPTL3, giving a direct connection to lipid metabolism (Haller *et al.* 2017). While we cannot rule out a role for *DOCK6*, these lines of evidence and a rare coding variant in *ANGPTL8* associated with HDL-C (Peloso *et al.* 2014) suggest *ANGPTL8* as the most likely target gene at this HDL-C GWAS locus.

The *ANGPTL8* HDL-C GWAS locus exhibited unusual characteristics in fine-mapping because the associated variants defined by LD *r*^2^ with the lead GWAS variants span a larger chromosomal region in African Americans than in Europeans, opposite of most loci in the genome (International HapMap Consortium 2003). Our haplotype association analyses showed that the LD differences are due to a haplotype with 20% frequency in African Americans from WHI that is essentially not observed in Finns from METSIM (0.02% frequency). At this locus, low-frequency haplotypes (frequency < 0.03; Table 1) distinguish *r*^2^ thresholds of 0.8 and 0.5 in Finns, and the *r*^2^ dependence on allele frequency suggests that a LD threshold of *r*^2^ > 0.8 for selecting candidate variants may be too restrictive and miss potentially functional variants. Here, we considered an initial set of candidate variants based on a more liberal threshold of *r*^2^ > 0.5, resulting in 42 total variants. We then tested nine variants predicted by MANTRA, CAVIAR, and PAINTOR and that overlapped regulatory datasets in functional assays. Seven of nine variants showed evidence of allelic differences (Figure 5), but only rs12463177 and rs17699089 showed differences consistent with the direction of the eQTL association. Of four additional variants that overlapped regulatory regions but were not predicted to be functional in fine-mapping assays, none showed evidence of regulatory activity. Taken together, these data suggest that the joint use of fine-mapping and regulatory overlap can successfully identify variants exhibiting allelic differences in functional assays.

The mechanisms by which the variants that showed allelic differences in functional assays may work in concert remains unclear. The effect on transcriptional activity of rs12463177 was modest and only significant in one cell type (Figure 4 and Figure S6 in File S1). This marginal effect observed in cell lines may not represent the physiological effect *in vivo*. The magnitude of effect of rs737337 was much greater (50-fold increased transcriptional activity), but did not show significant allelic differences, in contrast to a previous report (Schmidt *et al.* 2015). rs737337 exhibited strong liver-specific, allele-specific protein binding, but the specific transcription factor(s) remain unknown. One possible mechanism is that the regulatory region containing rs737337 is a strong enhancer that drives expression of *ANGPTL8*, but that the region containing rs12463177 is important for regulating allele-specific expression. Transcriptional regulators bound at the multiple variants may act together via chromosomal looping with the *ANGPTL8* promoter. Further experiments, especially *in vivo*, are needed to elucidate the precise roles and interactions of the seven variants that showed allelic differences in transcriptional activity and/or protein binding.

In this study, we identified *ANGPTL8* as the target gene at this HDL-C GWAS locus, determined regulatory drivers of tissue specificity, and combined fine-mapping approaches and regulatory overlap with experimental assays to identify variants that may contribute to the HDL-C GWAS signal at *ANGPTL8*. Identifying variants underlying GWAS loci contributes to a growing understanding of target genes, their direction of effect, and metabolic phenotypes. Our results are consistent with previously described results at other GWAS loci where multiple common regulatory variants act together (Corradin *et al.* 2014; He *et al.* 2015; Roman *et al.* 2015), and continued work on the *ANGPTL8* locus will further clarify the complex mechanism of these variants.

## Supplementary Material

Supplemental material is available online at www.g3journal.org/lookup/suppl/doi:10.1534/g3.117.300088/-/DC1.
