# Supplementary material for: Trans-ancestry Fine Mapping and Molecular Assays Identify Regulatory Variants at the ANGPTL8 HDL-C GWAS Locus
Source: G3 (Bethesda). 2017 Jul 28;7(9):3217–27. doi: 10.1534/g3.117.300088 (PMC5592946; doi:10.1534/g3.117.300088)
Supplement: Supplementary file 1 [file 3217FileS1.pdf]

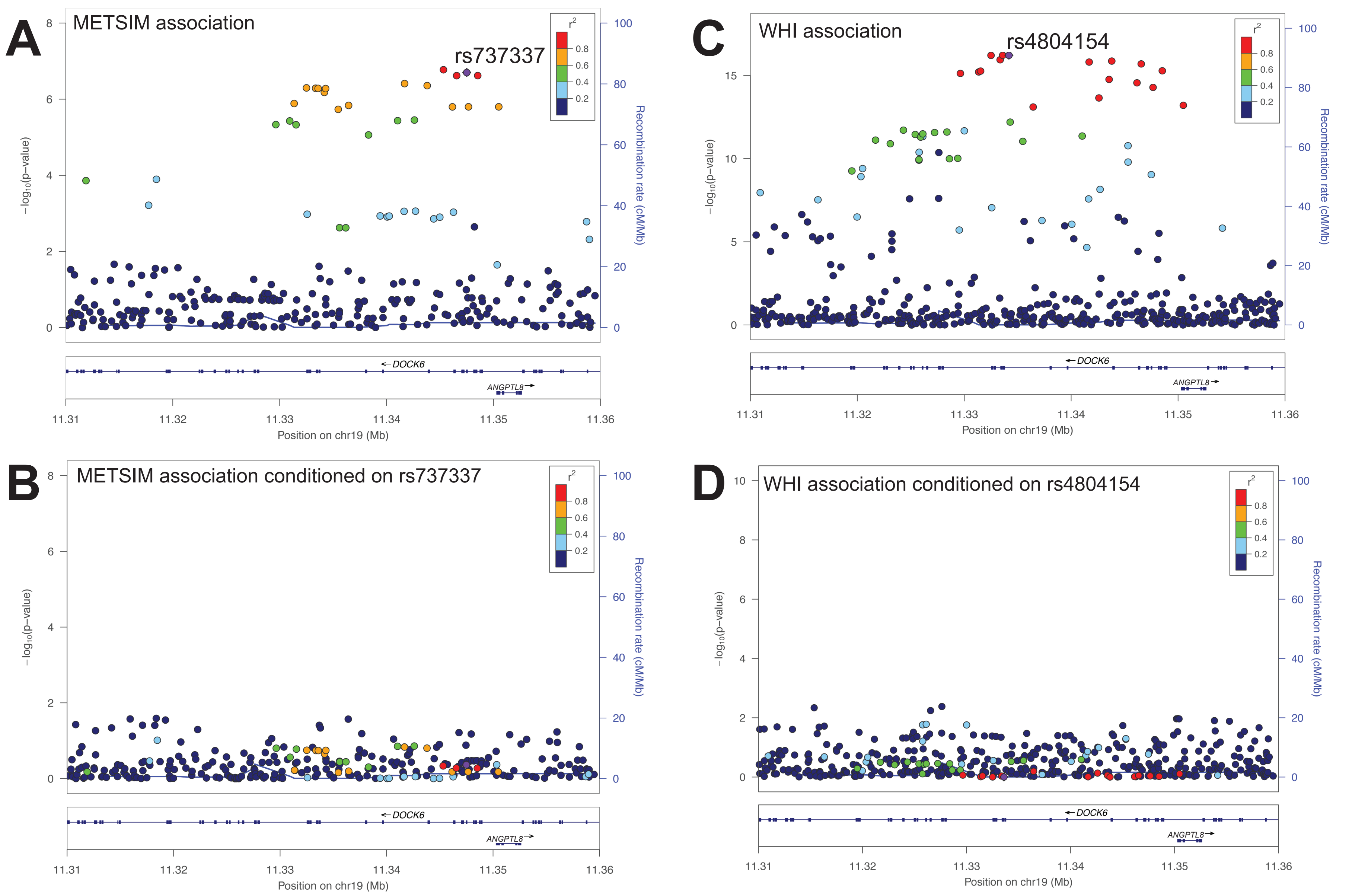

Figure S1. HDL association and conditional analysis

A: Variant association with concentration of phospholipids in medium HDL in the METSIM study of Finnish individuals (*N*=8380). rs737337 (purple) was among the most significantly associated variants. Variants are colored according to LD (r^2) with rs737337. B: Conditional analysis on rs737337 attenuated the association signal. C: HDL-C association in the WHI study of African American individuals (N=8244). Variants are colored according to LD (r^2) with rs4804154 (purple). D: Conditional analysis on rs4804154 attenuated the signal.

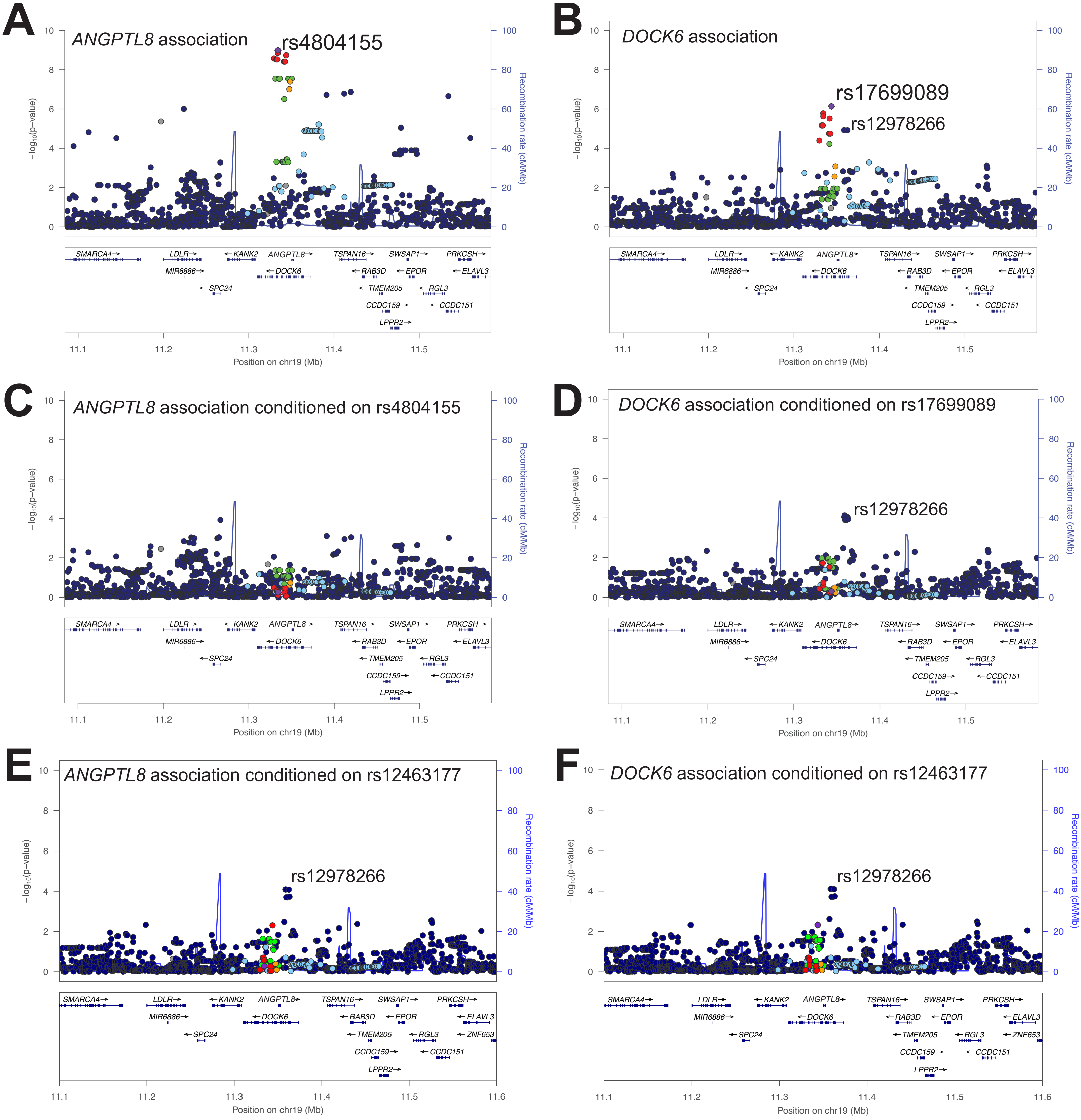

Figure S2. eQTL association in subcutaneous adipose from 770 individuals in the METSIM study
A: HDL-C-GWAS variants are associated with *ANGPTL8* expression. B: HDL-C GWAS variants are associated with *DOCK6* expression. C: Conditional analysis on the top variant associated with *ANGPTL8* expression, rs4804155, attenuated the signal. D: Conditional analysis on the top variant associated with *DOCK6* expression, rs17699089, revealed a secondary association signal with *DOCK6* expression (rs12978266, P=7.33x10-5). E: Conditional analysis on candidate functional variant rs12463177 also attenuated the *ANGPTL8* association signal and reveals the secondary association of rs12978266. F: Conditional analysis on candidate functional variant rs12463177 also attenuated the *DOCK6* association signal.

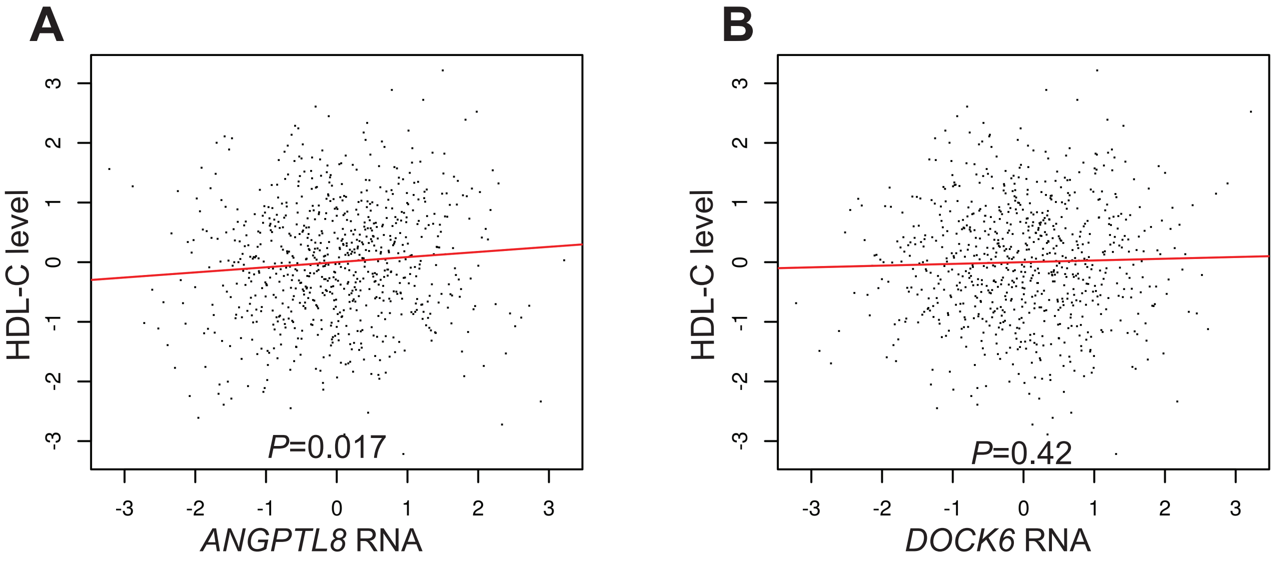

Figure S3. RNA associations with HDL-C in METSIM.
ANGPTL8 RNA levels (A) are associated with HDL-C level in 770 Finnish individuals from METSIM. *DOCK6* RNA levels (B) are not associated with HDL-C. To examine the relationship between RMA-normalized expression levels and HDL-C, we adjusted both traits for age and BMI, inverse normal transformed the residuals, and then tested for association in regression analysis. Correlation coefficients: R=0.086 (*ANGPTL8*), R=0.029 (*DOCK6*).

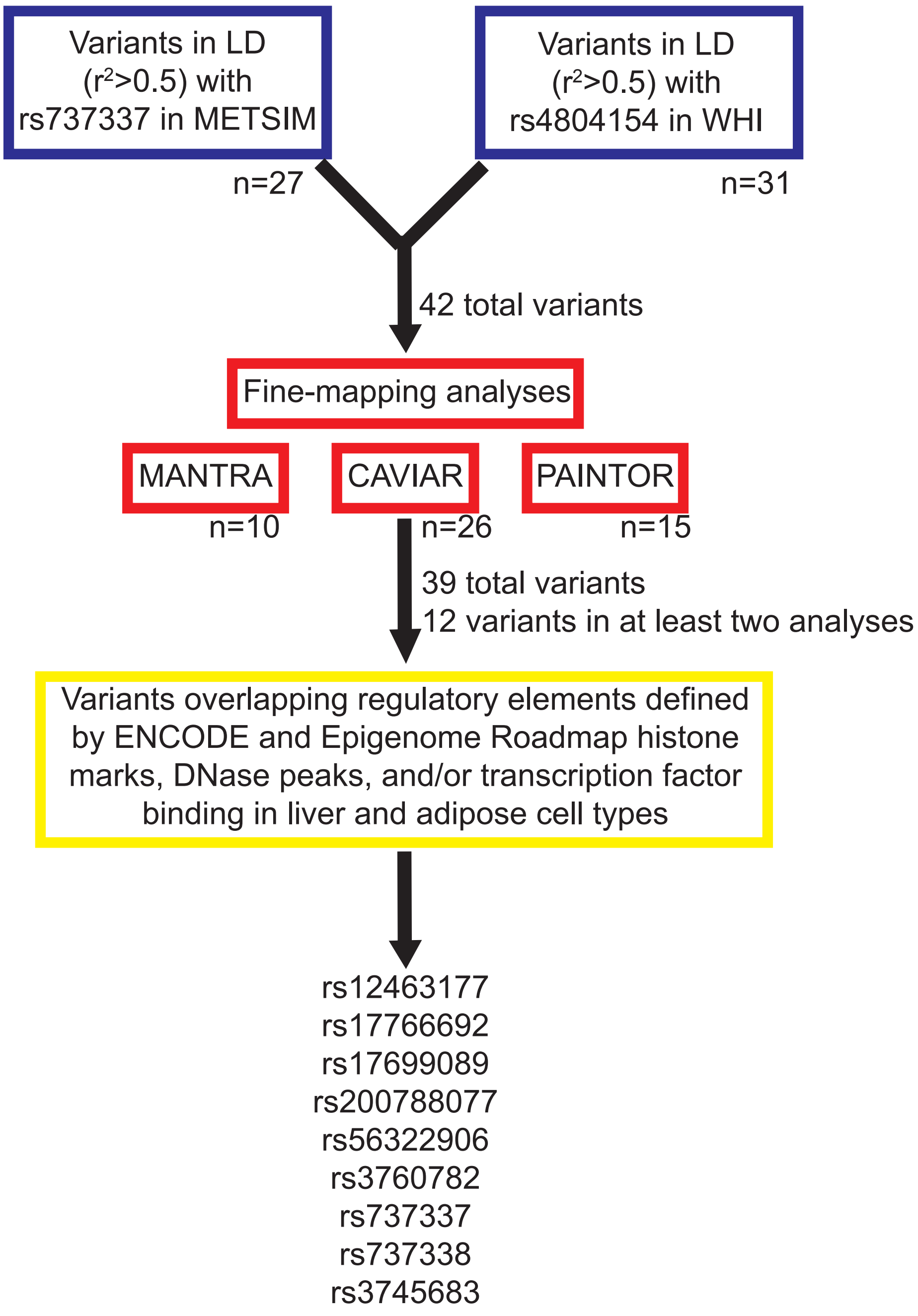

Figure S4. Flow chart describing selection of variants to test in functional experiments.

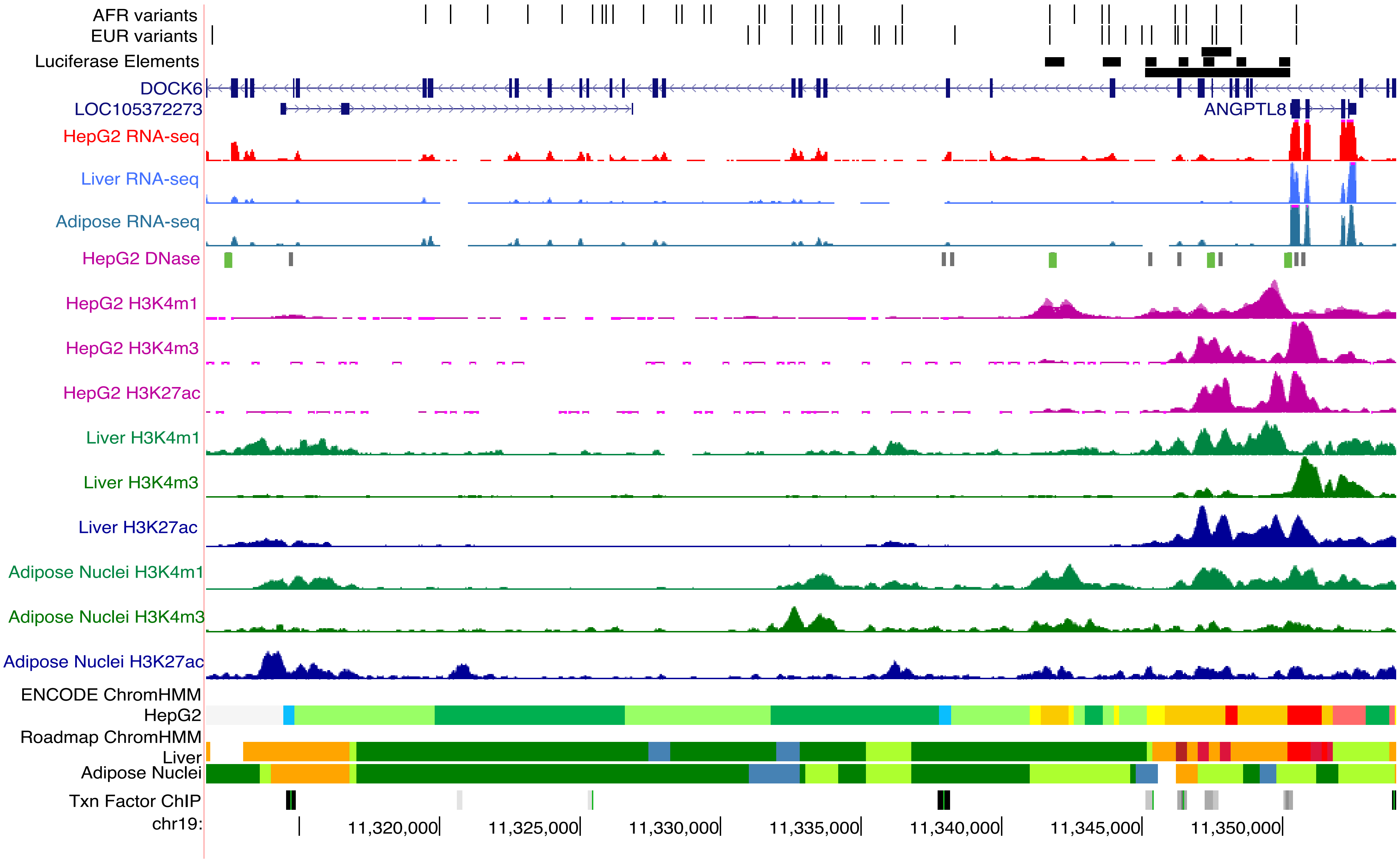

Figure S5. Candidate variants relative to predicted regulatory regions
27 variants exhibited r^2>0.5 with rs737337 in METSIM (EUR variants) and 31 variants with rs4804154 in WHI (AFR variants). These variants span a 39-kb window within *DOCK6*. 13 of 42 total variants overlap regulatory regions defined by histone marks, chromatin accessibility, and transcription factor binding (Figure 2). Green rectangles represent DNase hypersensitivity sites correlated with *ANGPTL8* expression.[26] Consistent with our tissue-specificity experiments, *ANGPTL8* is highly expressed in liver and adipose RNA-seq datasets.[24, 25] Data was accessed from ENCODE, the Epigenome Roadmap Atlas, and the UCSC Genome Browser. Black rectangles represent regions analyzed in transcriptional activity assays.

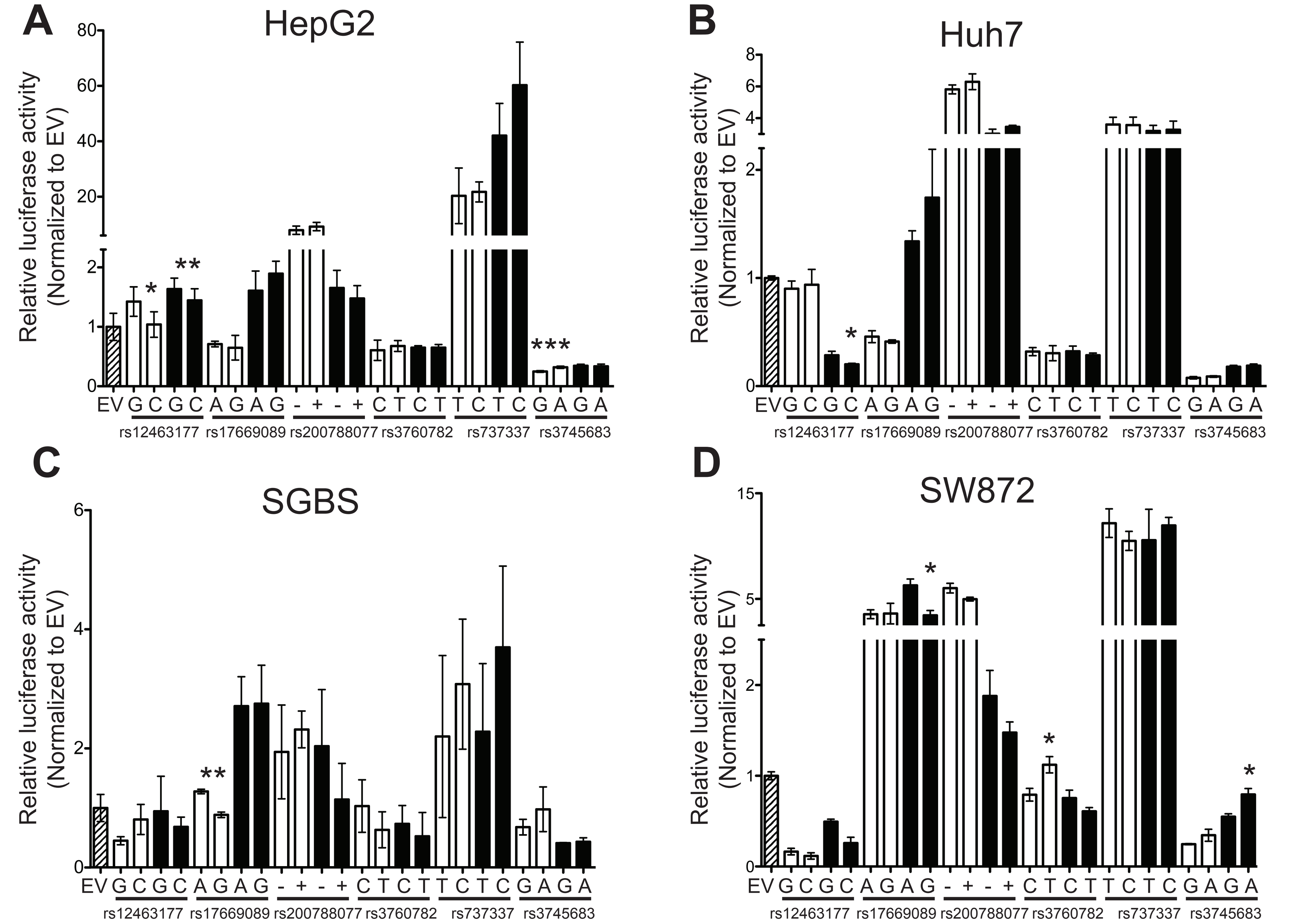

Figure S6. Variants tested in transcriptional reporter luciferase assays. Transcriptional activity was evaluated for six variants (rs56322906 was evaluated in a 5-kb haplotype, Fig S10) in HepG2 (A), Huh7 (B), SGBS (C), and SW872 (D) cells. Data are represented as the mean ± standard deviation of 3-5 biological replicates. Luciferase activity was normalized to empty vector (EV). White bars represent the forward orientation with respect to the genome; black are reverse. **P*<0.05, ***P*<0.01 ****P*<0.001 HDL-C-increasing alleles are presented first.

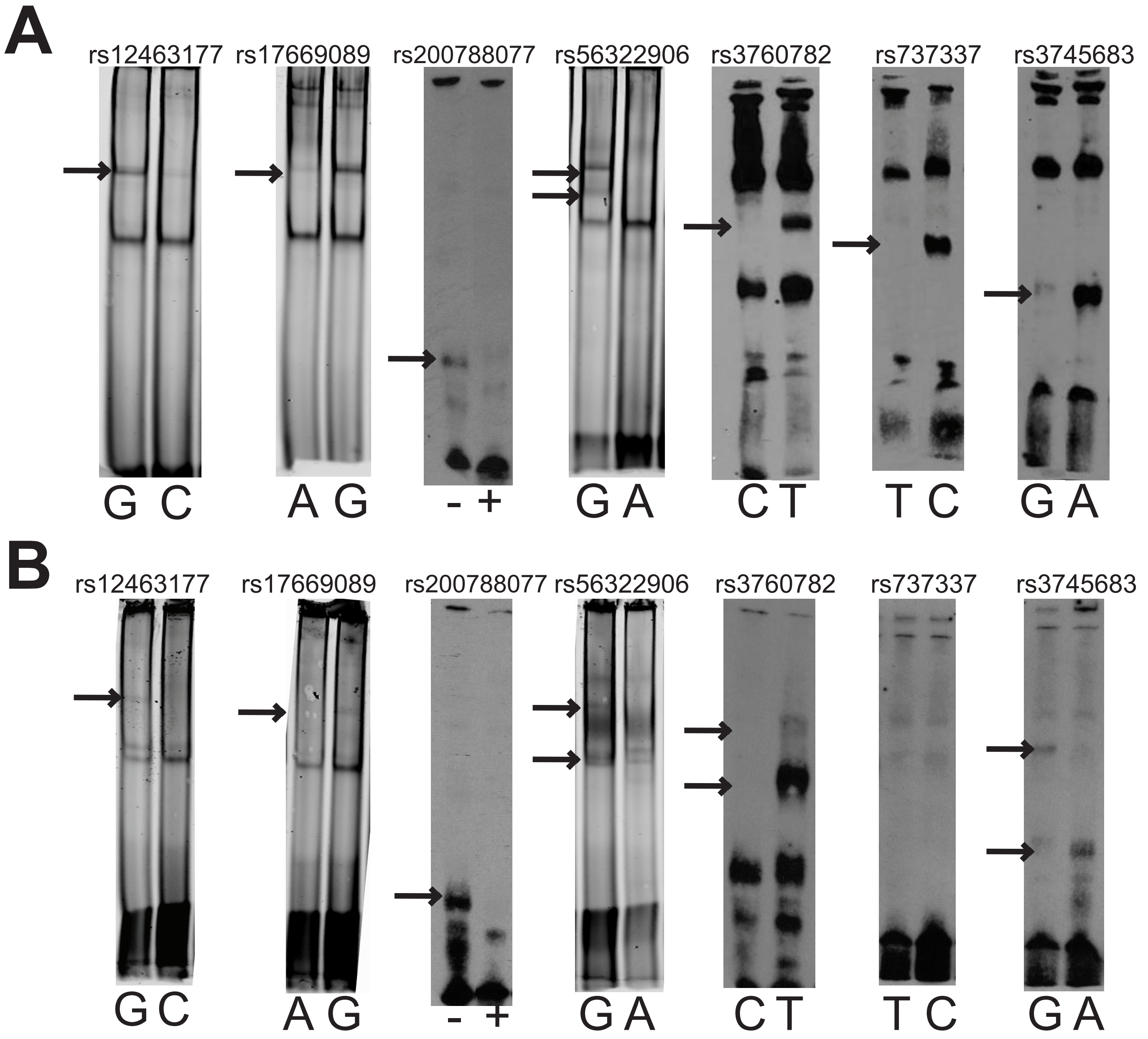

Figure S7. Seven variants show differential protein binding in EMSAs.
Allelic differences in protein binding were observed for all seven variants with nuclear extract from HepG2 cells (A). Six variants (except rs737337) showed allelic differences in protein binding with nuclear extract from SGBS cells (B). Arrows show allelic differences.

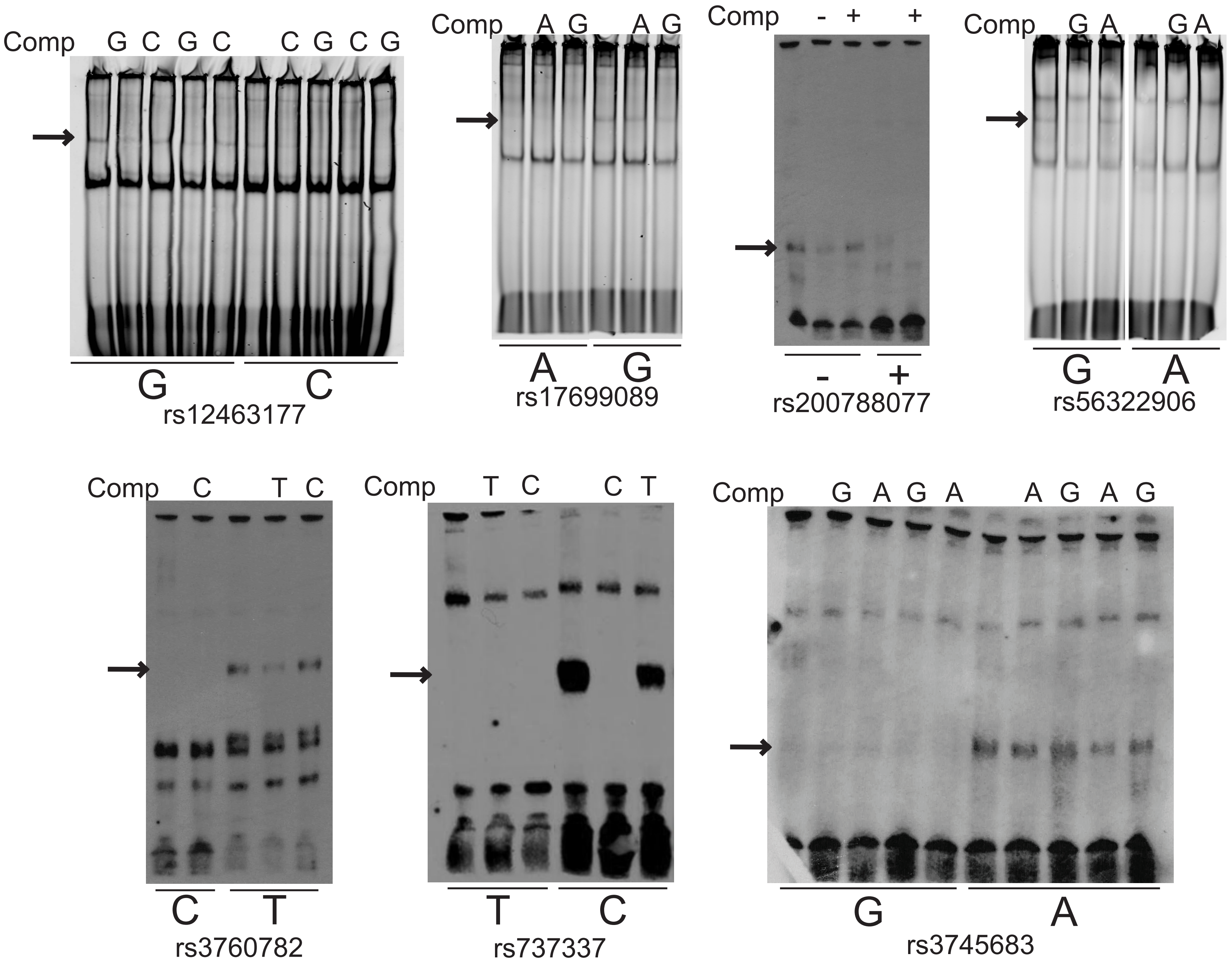

Figure S8. Competition EMSAs confirm allele-specific effects

Competition EMSA experiments using HepG2 nuclear extract were conducted with unlabeled competitor probes for each allele. rs12463177 is competed with 100x competition (lanes 2, 3, 7, 8) and 200x (lanes 4, 5, 9,10) compared to labeled probe. rs17699089 is competed with 100x competition, rs200788077 is competed with 100x competition, rs56322906 is competed with 100x competition, rs3760782 is competed with 192x competition, rs737337 is competed with 269x competition, and rs3745683 is competed with 50x (lanes 2, 3, 7, and 8) and 100x (lanes 4, 5, 9, and 10) competition. HDL-C-increasing alleles are presented first.

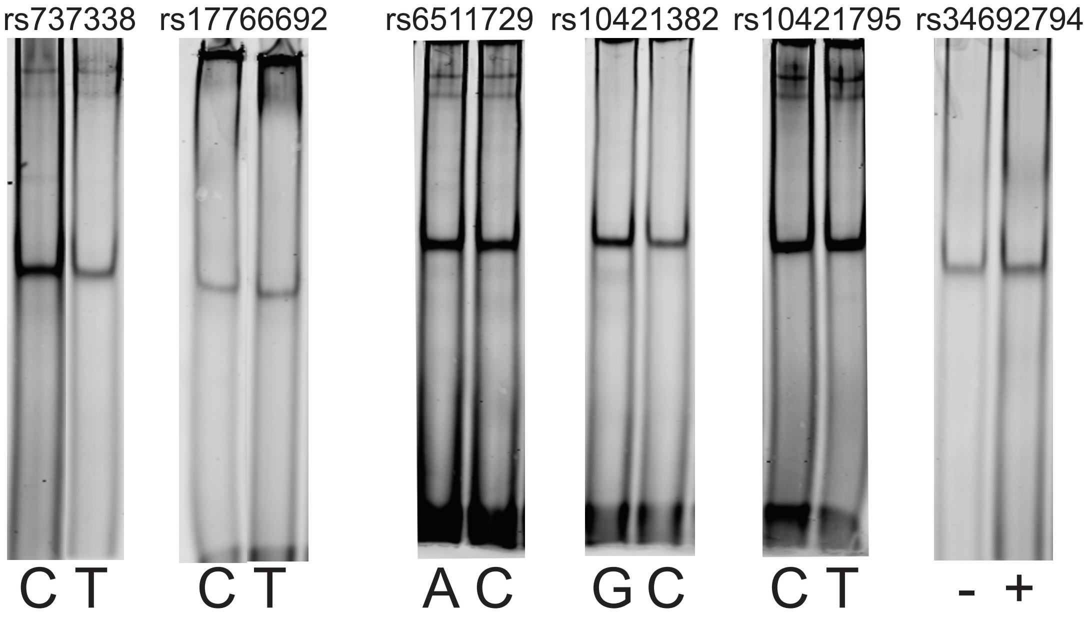

Figure S9. Six variants overlapping regulatory regions did not alter protein binding

EMSAs were performed with IR-labeled probes for each allele and incubated with 10 ug of HepG2 nuclear protein. rs737338 and rs17766692 were predicted in the CAVIAR causal set. The remaining four variants were not predicted in fine-mapping analyses. The prominent band in the middle of all gels represents non-specific binding that is observed in all IR-labeled EMSAs. HDL-C-increasing alleles are presented first. No allelic differences were observed.

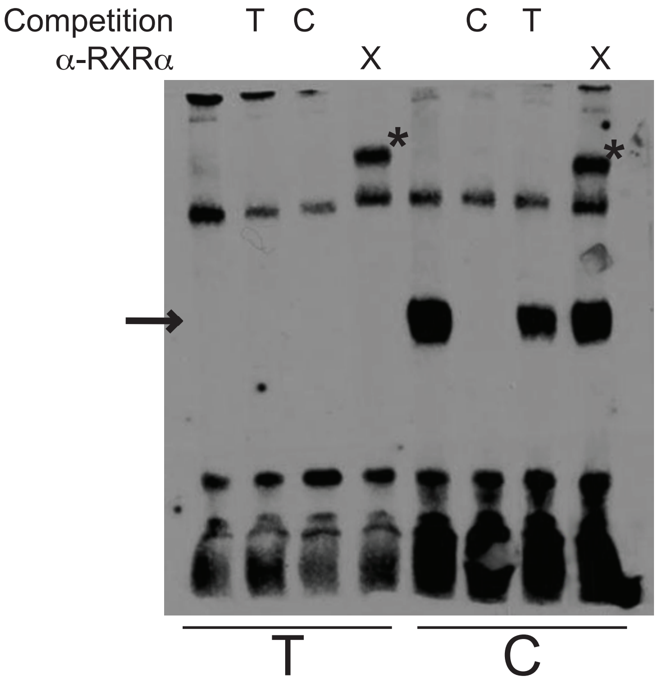

Figure S10. RXRα may bind in as part of a complex at rs737337
Supershift EMSA assays were performed using HepG2 nuclear extract (NE). The allele-specific band (arrow) is not disrupted when RXRα antibody is added to the reaction; however, there is a supershift in both alleles (asterisks). No disruption or supershift is observed with 36 other transcription factor antibodies (data not shown; Table S5). Competitor lanes are competed with 269x unlabeled probe. HDL-C-increasing alleles are presented first.

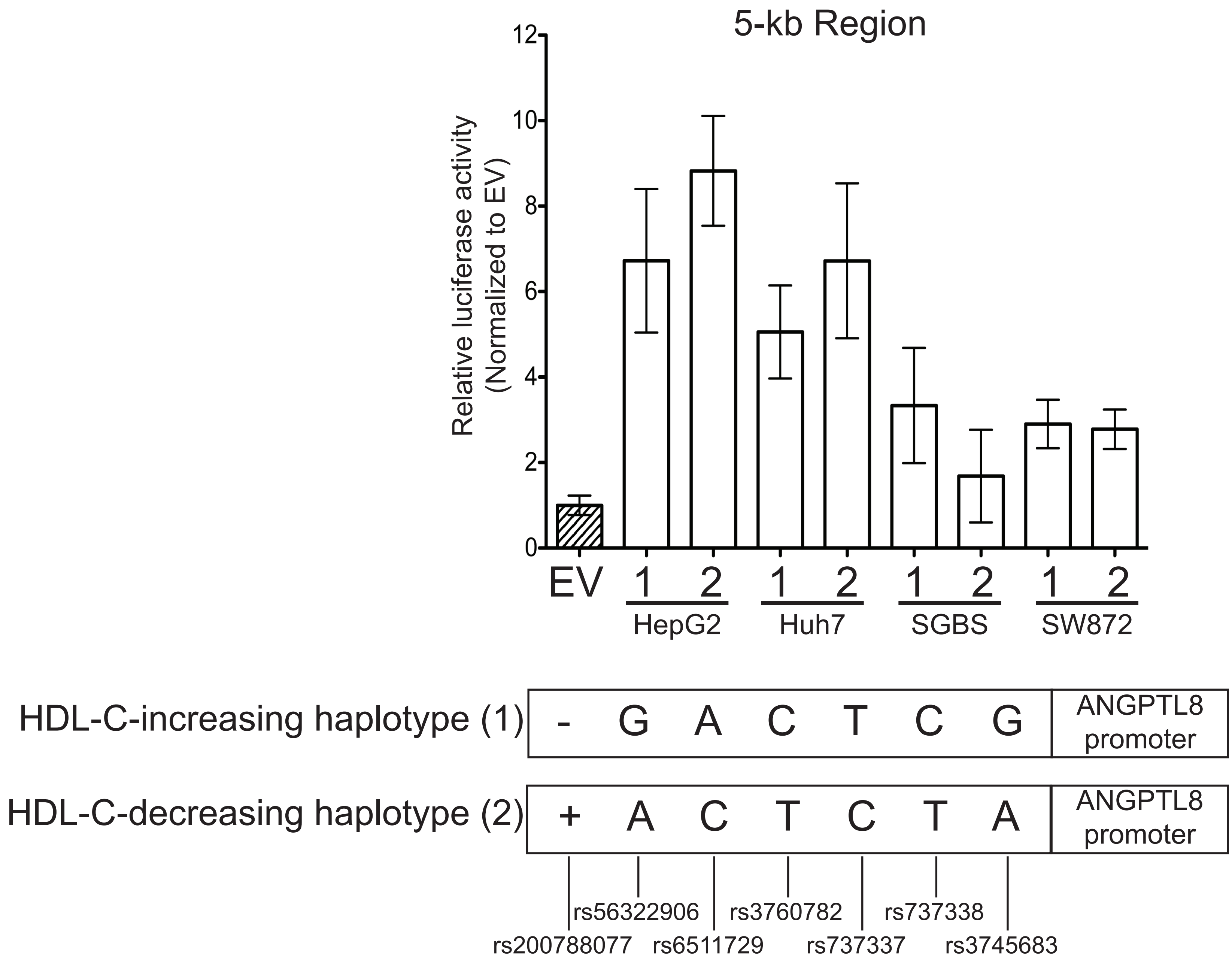

Figure S11. A 5-kb haplotype did not show allelic differences in transcriptional activity. Transcriptional activity was evaluated for a 5-kb haplotype containing 7 variants in HepG2, Huh7, SGBS, and SW872 cells. Data are represented as the mean ± standard deviation of 3-5 biological replicates. Luciferase activity was normalized to empty vector (EV). P>0.07 The HDL-C-increasing haplotype is haplotype 1.

Table S1. Primer and probe sequences for functional assays

Primer sequences for luciferase assays	5'- 3' Sequence	Chromosome Position (hg19)
rs737337_F<br>rs737337_R	gcaccagggtgaagaatttg<br>atcagtcagggagggctga	chr19:11347220-11347605
rs737337_long_F<br>rs737337_long_R	tcagcacagtgtccttgagc<br>tgctcacacccgatgtatgt	chr19:11347169-11348145
rs3745683_F<br>rs3745683_R	ctggcagctgacatggtaga<br>tatgtagggggacacgtgag	chr19:11348359-11348719
rs3760782_F<br>rs3760782_R	agtgccaggaaggcgaaag<br>aggtgacagtgagccgagat	chr19:11346293-11346640
rs200788077_F<br>rs200788077_R	ccctgagaataatgcctgaca<br>aatgttttgcccacttttgc	chr19:11345134-11345515
ANGPTL8prom_F<br>ANGPTL8prom_R	ggaggggaacaagagcagat<br>tctaaggtatagccacagcac	chr19:11349914-11350304
rs12463177_F<br>rs12463177_R	gctggtagggggtgagg<br>tgtgcttgagttagggctga	chr19:11341542-11342239
rs17699089_F<br>rs17699089_R	ttgttcagccacgccaag<br>cctggcctattctcagtttttc	chr19:11343636-11344234
5kb_F<br>5kb_R	ccctgagaataatgcctgaca<br>tctaaggtatagccacagcac	chr19:11345134-11350304
**Probe sequences for EMSA**	**5'- 3' Sequence**	
rs737337_T<br>rs737337_C	gacacggctgtgagctc<br>gacacggccgtgagctc	
rs3760782_C<br>rs3760782_T	aggggtcacaaattttt<br>aggggtcataaattttt	
rs200788077_+<br>rs200788077_-	aggaaaaaacaggctca<br>aggaaaaacaggctca	
rs3745683_G<br>rs3745683_A	tcacctctgccatgcca<br>tcacctctaccatgcca	
rs12463177_G<br>rs12463177_C	tgtgcaccgtgagggcc<br>tgtgcaccctgagggcct	
rs56322906_G<br>rs56322906_A	cgaactcctgacctcaaat<br>cgaactcctaacctcaaat	
rs6511729_A<br>rs6511729_C	ctcgtctcacagggatt<br>ctcgtctcccagggatt	
rs10421382_G<br>rs10421382_C	tgcctggcgtattttat<br>tgcctggcctattttat	
rs10421795_C<br>rs10421795_T	ccagttacctggggagg<br>ccagttacttggggagg	
rs17699089_A<br>rs17699089_G	tgtttcccatgcttcat<br>tgtttcccgtgcttcat	
rs34692794_-<br>rs34692794_G	atatgcatggggggtg<br>atatgcatgggggggtg	
rs17766692_C<br>rs17766692_T	tggatttgcacttcgtt<br>tggatttgtacttcgtt	
rs737338_C<br>rs737338_T	gtgtagcccggtctggg<br>gtgtagcctggtctggg	

Table S2. Association of 100 *ANGPTL8* locus variants with concentration of phospholipids in medium HDL in METSIM

Variant	Position	Alleles[a]	MAF	Unconditioned			Conditioned on rs737337		
				Effect	Std Error	P-value	Effect	Std Error	P-value
rs737337	19:11347493	T/C	0.08	-0.137	0.026	1.99E-07	-	-	-
rs112108870[b]	19:11345315	G/GA	0.08	-0.138	0.026	1.68E-07	-0.369	0.499	0.460
rs3745683	19:11348521	G/A	0.08	-0.137	0.026	2.39E-07	0.359	0.576	0.533
rs3760782	19:11346550	C/T	0.08	-0.137	0.026	2.40E-07	0.367	0.576	0.525
rs12463177	19:11341680	G/C	0.12	-0.115	0.023	3.88E-07	-0.056	0.039	0.146
rs17699089	19:11343795	A/G	0.12	-0.114	0.023	4.39E-07	-0.055	0.039	0.156
rs3826815	19:11332505	C/T	0.12	-0.114	0.023	5.03E-07	-0.053	0.039	0.178
rs72994363	19:11333358	G/T	0.12	-0.114	0.023	5.14E-07	-0.053	0.039	0.180
rs12974173	19:11333359	A/T	0.12	-0.114	0.023	5.16E-07	-0.052	0.039	0.180
rs3810308	19:11333596	T/C	0.12	-0.114	0.023	5.19E-07	-0.052	0.039	0.181
rs4804155	19:11334295	C/G	0.12	-0.114	0.023	5.24E-07	-0.052	0.039	0.179
rs4804154	19:11334179	C/T	0.12	-0.114	0.023	6.61E-07	-0.049	0.040	0.222
rs4804576	19:11331354	G/T	0.06	-0.142	0.029	1.30E-06	-0.033	0.063	0.599
rs66466742	19:11336444	C/T	0.06	-0.141	0.029	1.45E-06	-0.030	0.063	0.635
rs737338	19:11347657	C/T	0.06	-0.141	0.029	1.58E-06	-0.028	0.063	0.661
rs2278426	19:11350488	C/T	0.06	-0.141	0.029	1.58E-06	-0.028	0.063	0.661
rs56322906	19:11346155	G/A	0.06	-0.141	0.029	1.59E-06	-0.027	0.063	0.662
rs8101801	19:11335477	C/A	0.07	-0.139	0.029	1.84E-06	-0.025	0.062	0.689
rs17766692	19:11342599	C/T	0.10	-0.112	0.024	3.52E-06	-0.049	0.033	0.138
rs1865063	19:11341029	C/T	0.10	-0.112	0.024	3.65E-06	-0.049	0.033	0.140
rs17699030	19:11330942	A/G	0.05	-0.158	0.034	3.72E-06	-0.057	0.051	0.272
rs4804575	19:11329641	G/A	0.10	-0.110	0.024	4.63E-06	-0.046	0.033	0.157
rs4804153	19:11331531	C/T	0.10	-0.111	0.024	4.68E-06	-0.046	0.033	0.166
rs138572354	19:11338309	C/A	0.05	-0.149	0.033	8.66E-06	-0.036	0.053	0.496
rs143466522	19:11318472	G/A	0.02	-0.213	0.056	1.27E-04	-0.103	0.062	0.097
rs79846490	19:11311885	G/C	0.04	-0.146	0.038	1.38E-04	-0.023	0.052	0.661
rs111279811	19:11298369	C/T	0.03	-0.158	0.042	1.49E-04	-0.046	0.052	0.375
rs56048141	19:11317744	C/T	0.02	-0.173	0.051	6.10E-04	-0.056	0.058	0.342
rs12979813	19:11342703	A/G	0.18	-0.062	0.019	8.72E-04	-0.003	0.024	0.883
rs10406522	19:11341635	T/C	0.18	-0.062	0.019	8.81E-04	-0.003	0.024	0.886
rs6511729	19:11346252	A/C	0.18	-0.062	0.019	9.28E-04	-0.003	0.024	0.891
rs3810307	19:11332570	T/A	0.18	-0.061	0.019	1.05E-03	-0.002	0.024	0.941
rs2043302	19:11339396	T/C	0.18	-0.061	0.019	1.16E-03	-0.001	0.024	0.972
rs10418759	19:11340242	A/G	0.18	-0.061	0.019	1.18E-03	-0.001	0.024	0.976
rs7247404	19:11268556	G/A	0.34	0.050	0.015	1.22E-03	0.037	0.016	0.018
rs17001244	19:11340057	A/G	0.18	-0.060	0.019	1.23E-03	0.000	0.024	0.989
rs11672123	19:11194823	G/A	0.09	-0.080	0.025	1.24E-03	-0.075	0.025	0.002
rs10421382	19:11344973	G/C	0.18	-0.060	0.019	1.25E-03	-0.001	0.024	0.980
rs776487142	19:11013429	C/T	0.00	-5.925	1.842	1.31E-03	-5.973	1.840	0.001
rs10409274	19:11273179	G/A	0.29	0.051	0.016	1.34E-03	0.034	0.016	0.035
rs11671937	19:11264514	C/T	0.34	0.049	0.015	1.36E-03	0.036	0.016	0.020
rs7408517	19:11264063	C/T	0.34	0.049	0.015	1.39E-03	0.036	0.016	0.020
rs10421795	19:11344406	C/T	0.18	-0.060	0.019	1.39E-03	0.000	0.024	0.991
rs11670169	19:11266015	T/C	0.34	0.049	0.015	1.41E-03	0.036	0.016	0.020
rs892115	19:11263650	G/T	0.34	0.049	0.015	1.42E-03	0.036	0.016	0.020
rs934424	19:11267613	G/A	0.34	0.049	0.015	1.49E-03	0.036	0.016	0.021
rs9749459	19:11270016	T/C	0.29	0.050	0.016	1.55E-03	0.034	0.016	0.039

rs4804148	19:11270867	C/T	0.29	0.050	0.016	1.60E-03	0.034	0.016	0.040
rs9749257	19:11269960	G/T	0.29	0.050	0.016	1.61E-03	0.033	0.016	0.040
rs17248748	19:11206040	C/T	0.02	-0.175	0.056	1.62E-03	-0.110	0.057	0.056
rs4804579	19:11358700	T/C	0.18	-0.059	0.019	1.65E-03	0.004	0.024	0.882
rs8104261	19:11272585	C/T	0.29	0.050	0.016	1.71E-03	0.033	0.016	0.042
rs9749350	19:11257299	C/G	0.18	-0.059	0.019	1.98E-03	-0.044	0.019	0.023
rs934425	19:11267503	C/T	0.29	0.050	0.016	2.02E-03	0.042	0.016	0.011
rs551841239	19:11671580	G/C	0.00	-1.435	0.465	2.05E-03	-1.371	0.465	0.003
rs150205856	19:11391990	C/T	0.02	-0.180	0.059	2.18E-03	-0.062	0.065	0.338
rs200600677	19:11369440	TC/T	0.23	-0.053	0.017	2.19E-03	-0.022	0.019	0.240
rs17001264	19:11348212	C/T	0.02	-0.181	0.059	2.25E-03	-0.062	0.065	0.340
rs6511728	19:11335597	A/G	0.15	-0.062	0.020	2.37E-03	0.026	0.029	0.361
rs8101802	19:11336182	G/C	0.15	-0.062	0.020	2.38E-03	0.026	0.029	0.360
rs139606057	19:11393073	C/T	0.00	-1.089	0.359	2.40E-03	-1.100	0.358	0.002
rs112550373	19:11071469	G/A	0.01	0.277	0.091	2.41E-03	0.299	0.091	0.001
rs147629608	19:11411726	C/T	0.00	0.862	0.284	2.41E-03	0.953	0.284	0.001
rs34098	19:11539681	A/T	0.10	0.079	0.026	2.45E-03	0.057	0.027	0.031
rs186292971	19:11435015	A/C	0.00	1.608	0.531	2.47E-03	1.598	0.530	0.003
rs187416509	19:11528292	C/T	0.00	-1.387	0.462	2.66E-03	-1.414	0.461	0.002
rs145277768	19:11440420	C/T	0.00	1.595	0.532	2.69E-03	1.586	0.531	0.003
rs541868466	19:11543169	C/T	0.00	-1.384	0.462	2.73E-03	-1.411	0.461	0.002
rs181565096	19:11232702	C/G	0.02	-0.163	0.054	2.75E-03	-0.098	0.056	0.080
rs10423399	19:11273603	T/G	0.29	0.048	0.016	2.78E-03	0.031	0.016	0.057
rs565352617	19:11992491	CAA/C	0.00	-1.488	0.498	2.79E-03	-1.445	0.497	0.004
rs570249721	19:11252163	G/A	0.00	-9.531	3.225	3.13E-03	-9.358	3.221	0.004
rs191370629	19:11569128	C/T	0.00	-1.366	0.463	3.16E-03	-1.394	0.462	0.003
rs182210127	19:11659265	G/A	0.00	-1.352	0.458	3.16E-03	-1.377	0.457	0.003
rs112541805	19:11221946	G/GA	0.03	-0.128	0.045	4.12E-03	-0.087	0.045	0.054
rs386474655	19:11026074	G/C	0.00	-0.486	0.170	4.12E-03	-0.407	0.170	0.017
rs34254024	19:11369433	TTC/T	0.24	-0.049	0.017	4.14E-03	-0.019	0.018	0.311
rs13306513	19:11218226	G/A	0.03	-0.129	0.045	4.25E-03	-0.088	0.046	0.054
rs17242899	19:11216768	T/C	0.03	-0.129	0.045	4.25E-03	-0.088	0.046	0.054
rs145446845	19:11299431	G/A	0.02	-0.164	0.057	4.26E-03	-0.046	0.063	0.467
rs140898392	19:11218361	G/A	0.03	-0.129	0.045	4.28E-03	-0.088	0.046	0.055
rs146559752	19:11264537	A/C	0.03	0.118	0.042	4.42E-03	0.109	0.041	0.008
rs528191740	19:11448303	T/G	0.00	2.902	1.024	4.62E-03	2.889	1.023	0.005
rs200345643	19:11286646	G/C	0.00	-0.619	0.219	4.79E-03	-0.499	0.221	0.024
rs72996217	19:11358966	G/A	0.16	-0.055	0.020	4.81E-03	-0.007	0.023	0.764
rs11085768	19:11370653	G/A	0.24	-0.048	0.017	4.86E-03	-0.017	0.018	0.346
rs7248924	19:11372077	T/C	0.24	-0.047	0.017	5.55E-03	-0.017	0.018	0.352
rs142159985	19:11516368	A/C	0.00	2.395	0.865	5.63E-03	2.367	0.864	0.006
rs199636757	19:11378355	CAG/C	0.24	-0.047	0.017	5.89E-03	-0.017	0.018	0.363
.	19:11798665	A/G	0.00	-3.388	1.231	5.92E-03	-3.429	1.229	0.005
rs2043303	19:11368648	C/T	0.24	-0.047	0.017	5.98E-03	-0.017	0.018	0.366
rs7258016	19:11367353	A/C	0.24	-0.047	0.017	5.98E-03	-0.016	0.018	0.367
rs12462741	19:11365281	C/T	0.24	-0.047	0.017	6.02E-03	-0.016	0.018	0.369
rs2043301	19:11365650	C/A	0.24	-0.047	0.017	6.02E-03	-0.016	0.018	0.369
rs556896609	19:11444804	A/G	0.00	1.369	0.499	6.04E-03	1.358	0.498	0.006
rs322135	19:11379717	A/G	0.24	-0.047	0.017	6.08E-03	-0.016	0.018	0.368
rs7247840	19:11267678	T/C	0.26	0.046	0.017	6.09E-03	0.039	0.017	0.019
rs10422673	19:11265408	C/G	0.26	0.046	0.017	6.09E-03	0.039	0.017	0.019
rs396460	19:11374916	C/T	0.24	-0.047	0.017	6.10E-03	-0.016	0.018	0.368
rs416231	19:11374675	C/T	0.24	-0.047	0.017	6.11E-03	-0.016	0.018	0.369

Evidence of association with the concentration of phospholipids in medium HDL in 8,380 individuals in the METSIM study. Effect represents the change in standard-normalized residuals of phospholipids in medium HDL. Conditioning on variant rs737337 attenuated the signal. MAF, minor allele frequency

[a]Non-effect allele/effect allele

[b]rs112108870 is also known as rs200788077

Table S3. Association of 100 *ANGPTL8* locus variants with HDL-C in WHI

Variant	Position	NEA	EA	MAF	Unconditioned			Conditioned on rs48041545		
					Effect	Std Error	P-value	Effect	Std Error	P-value
rs4804154	19:11334179	C	T	0.19	-0.042	0.005	6.15E-17	-	-	-
rs3810308	19:11333596	T	C	0.19	-0.042	0.005	6.11E-17	0.000	0.005	0.999
rs3826815	19:11332505	C	T	0.19	-0.042	0.005	6.20E-17	0.000	0.005	0.996
rs12974173	19:11333359	A	T	0.17	-0.046	0.006	1.14E-16	-0.001	0.006	0.923
rs72994363	19:11333358	G	T	0.17	-0.046	0.006	1.14E-16	-0.001	0.006	0.923
rs17699089	19:11343795	A	G	0.20	-0.043	0.005	1.36E-16	0.000	0.005	0.998
rs12463177	19:11341680	G	C	0.20	-0.042	0.005	1.56E-16	0.000	0.005	0.985
rs3760782	19:11346550	C	T	0.19	-0.044	0.005	2.01E-16	-0.001	0.005	0.892
rs3745683	19:11348521	G	A	0.19	-0.044	0.005	5.22E-16	0.000	0.005	0.955
rs4804153	19:11331531	C	T	0.18	-0.044	0.005	5.43E-16	0.001	0.005	0.920
rs4804576	19:11331354	G	T	0.18	-0.043	0.005	6.14E-16	0.000	0.005	0.977
rs4804575	19:11329641	G	A	0.19	-0.043	0.005	7.50E-16	0.001	0.005	0.861
rs34692794	19:11343547	R	I	0.21	-0.041	0.005	1.72E-15	0.001	0.005	0.875
rs56322906	19:11346155	G	A	0.18	-0.043	0.005	2.76E-15	0.000	0.005	0.971
rs737338	19:11347657	C	T	0.18	-0.043	0.005	5.20E-15	0.001	0.005	0.917
rs17766692	19:11342599	C	T	0.20	-0.040	0.005	2.24E-14	0.002	0.005	0.736
rs2278426	19:11350488	C	T	0.19	-0.042	0.006	6.22E-14	0.002	0.006	0.780
rs66466742	19:11336444	C	T	0.17	-0.040	0.005	7.83E-14	0.002	0.005	0.640
rs4804155	19:11334295	C	G	0.31	-0.030	0.004	6.33E-13	-0.004	0.004	0.300
rs35472533	19:11324312	G	A	0.34	-0.032	0.004	1.93E-12	-0.005	0.004	0.313
rs62129150	19:11330005	G	A	0.52	0.030	0.004	2.12E-12	0.010	0.004	0.017
rs113441245	19:11328383	G	A	0.35	-0.031	0.004	2.50E-12	-0.004	0.004	0.356
rs11085764	19:11327227	G	C	0.35	-0.031	0.004	2.68E-12	-0.004	0.004	0.350
rs2304154	19:11326125	C	T	0.35	-0.031	0.004	3.22E-12	-0.004	0.004	0.357
rs2163830	19:11325417	A	G	0.35	-0.031	0.004	3.51E-12	-0.004	0.004	0.359
rs1865063	19:11341029	C	T	0.31	-0.030	0.004	4.35E-12	-0.005	0.004	0.256
rs2304155	19:11326119	G	A	0.52	0.030	0.004	4.72E-12	0.010	0.004	0.017
rs11673129	19:11325924	C	G	0.35	-0.031	0.004	5.06E-12	-0.004	0.004	0.375
rs8113156	19:11321705	T	G	0.35	-0.030	0.004	7.63E-12	-0.004	0.004	0.317
rs8101801	19:11335477	C	A	0.30	-0.029	0.004	9.08E-12	-0.005	0.004	0.278
rs11666686	19:11323085	T	C	0.35	-0.030	0.004	1.26E-11	-0.004	0.004	0.387
rs112108870	19:11345315	R	I	0.40	-0.029	0.004	1.67E-11	-0.008	0.004	0.053
rs2116873	19:11325784	A	T	0.55	0.030	0.004	4.11E-11	0.008	0.004	0.062
rs4804152	19:11327626	G	A	0.39	0.030	0.004	4.28E-11	0.013	0.004	0.004
rs59389322	19:11329394	G	A	0.34	-0.029	0.005	9.54E-11	-0.002	0.004	0.578
rs67076391	19:11328617	C	T	0.34	-0.029	0.005	1.01E-10	-0.002	0.004	0.582
rs2116875	19:11325764	A	G	0.30	-0.032	0.005	1.13E-10	-0.001	0.005	0.777
rs2116874	19:11325767	T	C	0.43	-0.030	0.005	1.23E-10	-0.011	0.005	0.017
rs200788077	19:11345320	R	I	0.37	-0.028	0.004	1.60E-10	-0.009	0.004	0.051
rs111705028	19:11320494	C	T	0.25	-0.036	0.006	3.91E-10	-0.007	0.006	0.215
rs8409	19:11319491	G	A	0.37	-0.027	0.004	5.46E-10	-0.003	0.004	0.515
rs737337	19:11347493	T	C	0.41	-0.026	0.004	9.09E-10	-0.006	0.004	0.169
rs12981155	19:11320339	G	C	0.27	-0.036	0.006	1.20E-09	-0.006	0.006	0.309
rs12979813	19:11342703	A	G	0.50	-0.022	0.004	7.01E-09	-0.006	0.004	0.102
rs7252965	19:11309160	G	C	0.53	0.024	0.004	8.49E-09	0.006	0.004	0.149
rs8101345	19:11310920	T	C	0.45	-0.023	0.004	1.10E-08	-0.005	0.004	0.195
rs4804151	19:11327608	C	T	0.36	0.026	0.005	2.44E-08	0.010	0.005	0.033
rs12609620	19:11324890	C	T	0.36	0.026	0.005	2.58E-08	0.010	0.005	0.031
rs10406522	19:11341635	T	C	0.51	-0.022	0.004	2.62E-08	-0.006	0.004	0.138
rs8110433	19:11316317	A	C	0.55	0.023	0.004	2.97E-08	0.005	0.004	0.208
rs138111115	19:11307572	R	D	0.50	0.023	0.004	4.66E-08	0.006	0.004	0.171

rs3810307	19:11332570	T	A	0.49	-0.022	0.004	8.85E-08	-0.004	0.004	0.277
rs149928810	19:11308475	R	D	0.49	0.022	0.004	2.24E-07	0.005	0.004	0.255
rs764304127	19:11314807	D	R	0.46	0.021	0.004	2.27E-07	0.006	0.004	0.166
rs11878417	19:11319978	A	G	0.52	0.021	0.004	3.19E-07	0.002	0.004	0.615
rs10421795	19:11344406	C	T	0.62	-0.022	0.004	3.30E-07	-0.008	0.004	0.075
rs113535966	19:11337269	G	A	0.06	-0.051	0.01	5.22E-07	-0.002	0.01	0.822
rs10421382	19:11344973	G	C	0.61	-0.021	0.004	5.61E-07	-0.007	0.004	0.100
rs6511728	19:11335597	A	G	0.61	-0.021	0.004	5.95E-07	-0.007	0.004	0.086
rs7252976	19:11315343	G	A	0.20	0.025	0.005	6.36E-07	0.014	0.005	0.005
rs17001244	19:11340057	A	G	0.54	-0.019	0.004	8.72E-07	-0.004	0.004	0.298
rs2043302	19:11339396	T	C	0.61	-0.021	0.004	1.09E-06	-0.006	0.004	0.162
rs35248735	19:11312238	G	A	0.42	0.019	0.004	1.27E-06	0.005	0.004	0.181
rs56865998	19:11354146	C	G	0.07	-0.047	0.01	1.49E-06	-0.002	0.01	0.872
rs59175057	19:11329534	R	D	0.44	-0.021	0.004	1.94E-06	-0.004	0.004	0.368
rs184781818	19:11317340	A	G	0.00	-1.927	0.413	2.99E-06	-0.492	0.411	0.231
rs114281937	19:11348208	C	T	0.02	-0.084	0.018	3.04E-06	-0.034	0.018	0.060
.	19:11323240	D	R	0.38	0.020	0.004	3.29E-06	0.005	0.004	0.243
rs7249565	19:11302807	G	A	0.60	0.018	0.004	3.44E-06	0.004	0.004	0.300
rs12980863	19:11309871	C	T	0.41	0.018	0.004	3.52E-06	0.005	0.004	0.214
rs3745681	19:11303943	A	G	0.59	0.018	0.004	3.63E-06	0.003	0.004	0.447
rs7246614	19:11310538	C	T	0.24	0.021	0.005	3.87E-06	0.010	0.005	0.024
rs3745682	19:11313256	G	A	0.24	0.021	0.005	4.20E-06	0.010	0.004	0.025
rs4804574	19:11317482	A	G	0.35	0.019	0.004	4.55E-06	0.007	0.004	0.118
rs7250652	19:11302606	G	A	0.40	-0.018	0.004	4.63E-06	-0.004	0.004	0.255
rs145352947	19:11306346	R	D	0.58	0.019	0.004	4.86E-06	0.003	0.004	0.478
rs6511727	19:11315817	G	T	0.23	0.021	0.005	4.97E-06	0.011	0.005	0.021
rs10421221	19:11316547	T	C	0.32	0.019	0.004	6.41E-06	0.007	0.004	0.080
rs10418759	19:11340242	A	G	0.64	-0.019	0.004	6.54E-06	-0.005	0.004	0.196
rs8101802	19:11336182	G	C	0.62	-0.019	0.004	8.49E-06	-0.006	0.004	0.187
rs8110823	19:11316315	G	A	0.23	0.021	0.005	8.52E-06	0.011	0.005	0.019
.	19:11323239	D	R	0.38	0.019	0.004	9.08E-06	0.004	0.004	0.341
rs7250778	19:11306265	G	A	0.21	0.023	0.005	1.50E-05	0.012	0.005	0.018
rs61045132	19:11303068	C	T	0.22	0.020	0.005	1.73E-05	0.013	0.005	0.005
rs58543390	19:11342434	C	T	0.00	-1.187	0.279	2.07E-05	-0.328	0.278	0.237
rs73506605	19:11307564	G	A	0.44	0.018	0.004	2.10E-05	0.003	0.004	0.442
rs34757881	19:11341462	C	T	0.07	-0.039	0.009	2.18E-05	0.012	0.009	0.190
rs200384092	19:11323225	R	D	0.41	-0.023	0.005	2.93E-05	-0.006	0.005	0.258
rs79846490	19:11311885	G	C	0.01	-0.115	0.028	3.71E-05	-0.030	0.028	0.280
rs6511729	19:11346252	A	C	0.65	-0.018	0.004	3.72E-05	-0.004	0.004	0.336
rs57681847	19:11300648	G	T	0.21	0.019	0.005	4.02E-05	0.012	0.005	0.009
rs58495388	19:11300312	G	C	0.18	0.021	0.005	6.76E-05	0.014	0.005	0.008
rs139048611	19:11321312	R	D	0.02	-0.067	0.017	7.34E-05	-0.023	0.017	0.167
rs8111456	19:11301147	A	G	0.22	0.018	0.005	7.44E-05	0.013	0.005	0.007
rs147045092	19:11300357	G	C	0.00	14.200	3.656	0.000103	10.115	3.641	0.005
rs34301174	19:11348098	G	A	0.11	0.034	0.009	0.000115	0.017	0.009	0.054
rs2278013	19:11305429	C	A	0.15	0.024	0.006	0.000168	0.014	0.006	0.032
rs148312284	19:11358858	G	C	0.02	-0.071	0.019	0.000191	-0.025	0.019	0.182
rs199653227	19:11317508	D	R	0.25	0.017	0.005	0.000234	0.007	0.005	0.134
rs73506665	19:11358644	C	T	0.05	-0.036	0.01	0.000263	0.000	0.01	0.970

Evidence of association with HDL-C in 8,244 individuals in the WHI study. Effect represents the change in standard-normalized residuals of phospholipids in medium HDL. Conditioning on lead variant rs4804154 attenuated the signal. NEA, non-effect allele; EA, effect allele; MAF, minor allele frequency

Table S4. Variant associations with gene expression levels in subcutaneous adipose tissue.

	eQTL p-value					
**Gene**	**rs4804155**	**rs17699089**	**rs4804154**	**rs737337**	**rs12463177**	**Probeset ID**
ANGPTL8	1.04E-09	1.84E-09	1.38E-09	9.74E-08	3.84E-09	11756040_a_at
DOCK6	2.37E-06	7.21E-07	1.65E-06	2.68E-03	3.06E-06	11719400_a_at
CCDC159	0.071	0.066	0.085	0.092	0.054	11744239_a_at
KANK2	0.056	0.073	0.048	0.565	0.116	11726538_x_at
KRI1	0.052	0.062	0.036	0.348	0.053	11725949_x_at
LDLR	0.303	0.300	0.258	0.616	0.349	11720028_x_at
LPPR2	0.059	0.056	0.054	0.215	0.070	11722506_a_at
S1PR5	0.152	0.202	0.157	0.014	0.247	11752664_a_at
SLC44A2	0.028	0.029	0.028	0.178	0.036	11740973_s_at
TSPAN16	0.050	0.032	0.043	0.288	0.034	11761297_x_at

Gene expression was measured in 770 subcutaneous adipose samples. eQTL data are reported for genes within 1 Mb of rs737337 that have at least one variant with p<0.05. Lead eQTL variants for *ANGPTL8* (rs4804155) and *DOCK6* (rs17699089), lead GWAS variants (rs4804154 and rs737337) and functional candidate variant rs12463177 are shown. Data are shown for the most strongly associated probeset.

Table S5. Variants associated with HDLw C in METSIM and/or WHI

SNP[a]	chr19 position	r^2 with rs737337 (EUR)[c]	r^2 with rs4804154 (AFR)[c]	Dnased	FAIRE[d]	H3K4me1[d]	H3K27ac[d]	H3K4me3[d]	H3K9ac[d]	H3K4me2[d]	Transcription Factor Binding (ChIP-seq)[d]	Posterior Probability MANTRA	Posterior Probability CAVIAR (Finnish)[e]	Predicted in at least one PAINTOR analysis[f]	Antibodies tested in EMSA
rs79846490	11311884	0.58				L									
rs8409	11319491		0.66	NM											
rs8113156	11321705		0.72								B: ZEB1			Yes	
rs11666686	11323085		0.78												
rs35472533	11324312		0.81												
rs2163830	11325417		0.8												
rs2116875	11325764		0.68												
rs11673129	11325924		0.8												
rs2304154	11326125		0.82												
rs11085764	11327227		0.82											Yes	
rs113441245	11328383		0.78												
rs67076391	11328617		0.78												
rs59389322	11329394		0.79												
rs4804575	11329641	0.34	0.98									0.240	0.021		
rs17699030	11330942	0.45											0.029	Yes	
rs4804576	11331354	0.46	0.95									0.450	0.037		
rs4804153	11331531	0.3	0.95									0.240			
rs3826815	11332505	0.67	0.99					A					0.050	Yes	
rs12971537	11333358							A							
rs12974173	11333359	0.61	0.81					A					0.049		
rs3810308	11333596	0.67	0.98					A				0.290	0.049	Yes	
rs4804154	11334179	0.67	1								L: USF1		0.037		
rs4804155	11334295	0.67	0.48										0.050	Yes	
rs8101801	11335477	0.46	0.47										0.024		
rs6511728	11335597	0.48													
rs8101802	11336182	0.53					A								
rs66466742	11336444	0.46	0.92				A						0.028		
rs138572354	11338309	0.72		ONEBL									0.023		
**rs12463177**	11341680	0.74	0.93			AL	AL			L		0.320	0.065	Yes	
**rs17766692**	11342599	0.34	0.84			AL	L			L	B: OCT2, POU2f2		0.028		
**rs34692794**	11343547	0.74	0.86			AL									
**rs17699089**	11343795	0.74	0.91			AL	A					0.290	0.061	Yes	
**rs10421795**	11344406	0.47				AL									
**rs10421382**	11344973	0.47				L	A								
**rs200788077**[b]	11345321	0.96	0.22	L		L	A				L: JUND, cJUN		0.066		SMAD4, HNF4G, RXRA
**rs56322906**	11346155	0.54	0.86			AL	AL	L	L	L		0.420	0.026		
**rs6511729**	11346252	0.48		M		AL	AL	L	L	L					
**rs3760782**	11346550	0.98	0.87			AL	AL	L	L	L	L:HDAC2	0.430	0.051	Yes	SP1, RXRA, HNF4A, HNF4G, SREBP1, SREBP2
**rs737337**	11347493	1	0.26	L		AL	AL	L	L	L	L: USF1, TAF1, RXRA, ELF1		0.058		RXRA, AP2A, HIF1A, CEBPB, LXRA, CHREBP, THR, SP1, USF1, NF1, PAX4, FOXA1, FOXA2, FOXO3, PPARG, CEBPB, YY1, PAX6, PARP1, SMAD4, HEY1, FOSL1, ELF1, TAF1, HNF4G, SREBP1, SREBP2, CEBPA, AHR, ARNT, PPARA, EGR1, CREB, HNF4A, PXR, LRH1, CAR
**rs737338**	11347657	0.54	0.86			AL	AL	L	L	L	L: CEBPb, TAF1, ELF1, HNF4g	0.420	0.026		
**rs3745683**	11348521	0.98	0.82	N		AL	AL	L	L	L		0.420	0.051		YY1, SREBP1, RXRA, PPARG
rs2278426	11350488	0.54	0.78	L		AL	AL	L	L	L	L: POL2		0.026		

[a]Variants are ordered by position, hg19. Nearby genes: *ANGPTL8* is located at position 11350295-11352619, *DOCK6* position 11309969-11373168. The thirteen candidate variants evaluated in functional assays are bolded.

[b]rs200788077 is also known as rs112108870

[c]r^2-Haploreg v4.1 (1000 Genomes Phase I)

[d]DNase, FAIRE, histone marks, and ChIP-seq data are from ENCODE and Roadmap Epigenomics

[e]Three additional variants are identified in the Finnish CAVIAR analysis and two in the African American CAVIAR analysis that are not in LD with either lead.

[f]Nine additional variants are identified in at least one PAINTOR analysis that are not in LD with either lead.

O=Bone: ENCODE Osteoblast

B= Blood: ENCODE GM19240, GM19239, GM19238, GM190999, GM18951, GM18526, GM18507, GM15510, GM12892, GM12891, GM12878, GM12875, GM12874, GM12873, GM12872, GM12865, GM12864, GM12802, GM12193, GM06990

L=Liver: Roadmap Epigenomics Adult Liver; ENCODE Hepatocytes, HepG2, Huh-7

E=Endothelial: ENCODE HUVEC

M=Muscle: Roadmap Epigenomics Skeletal Muscle; ENCODE Myocyte, PSOAS muscle

N=Brain: Roadmap Epigenomics Brain Anterior Caudate, Brain Mid Frontal Lobe, Brain Substantia Nigra; ENCODE Astrocytes, Cerebellum, Cerebral Frontal, Frontal Cortex

A=Adipose: Roadmap Epigenomics Adipose Nuclei, Adipose Tissue

Table S6. Fine-mapping analysis using MANTRA

Variant	Position	EA	NEA	logBF	PP	Direction	Bayes Factor	Cum(BF)	credible set
rs3760782	11,346,550	T	C	18.9	0.43	--	7.57E+18	7.57E+18	0.331
rs3745683	11,348,521	A	G	18.8	0.42	--	6.04E+18	1.36E+19	0.595
rs4804153	11,331,531	T	C	18.6	0.24	--	3.72E+18	1.73E+19	0.757
rs17699089	11,343,795	G	A	18.4	0.29	--	2.28E+18	1.96E+19	0.856
rs4804576	11,331,354	T	G	17.9	0.45	--	7.56E+17	2.04E+19	0.889
rs56322906	11,346,155	A	G	17.8	0.42	--	6.60E+17	2.10E+19	0.918
rs4804575	11,329,641	A	G	17.8	0.24	--	6.48E+17	2.17E+19	0.947
rs737338	11,347,657	T	C	17.8	0.42	--	5.95E+17	2.23E+19	0.973
rs3810308	11,333,596	C	T	17.5	0.29	--	3.14E+17	2.26E+19	0.986
rs12463177	11,341,680	C	G	17.3	0.32	--	2.00E+17	2.28E+19	0.995

MANTRA analysis was conducted in 16624 individuals from METSIM and WHI. Credible set values are the cum(BF) divided by the total cumulative Bayes Factor. EA, effect allele; NEA, non-effect allele; logBF, log(Bayes Factor); PP, posterior probability

Table S7. Fine-mapping analysis using CAVIAR

Finnish		African American	
**Posterior probability**	**Variant**	**Posterior probability**	**Variant**
0.066	rs200788077	0.499	rs2116874
0.065	rs12463177	0.499	rs2304155
0.061	rs17699089		
0.058	rs737337		
0.051	rs3745683		
0.051	rs3760782		
0.050	rs3826815		
0.050	rs4804155		
0.049	rs72994363		
0.049	rs12974173		
0.049	rs3810308		
0.037	rs4804154		
0.031	rs4804576		
0.029	rs17699030		
0.028	rs66466742		
0.028	rs143466522		
0.028	rs17766692		
0.027	rs1865063		
0.026	rs737338		
0.026	rs2278426		
0.026	rs56322906		
0.024	rs8101801		
0.023	rs138572354		
0.021	rs4804575		

Variants shown are in the 95% causal set.

Table S8. Fine-mapping analysis using PAINTOR

METSIM Finnish

2 causal variants			3 causal variants			4 causal variants			5 causal variants		
Position	Variant	Probability	Position	Variant	Probability	Position	Variant	Probability	Position	Variant	Probability
11327571	rs4804150	0.42	11327571	rs4804150	1.00	11327571	rs4804150	1.00	11327571	rs4804150	1.00
11341680	rs12463177	0.29	11313256	rs3745682	1.00	11313256	rs3745682	0.96	11317770	rs56034303	1.00
11343795	rs17699089	0.29	11343795	rs17699089	0.66	11318375	rs116504889	0.86	11313256	rs3745682	0.97
11330942	rs17699030	0.25	11332505	rs3826815	0.34	11343795	rs17699089	0.65	11318375	rs116504889	0.88
11332505	rs3826815	0.23				11332505	rs3826815	0.30	11343795	rs17699089	0.62
11346550	rs3760782	0.20							11332505	rs3826815	0.26
									11318235	rs114277401	0.11

WHI African American

2 causal variants			3 causal variants			4 causal variants			5 causal variants		
Position	Variant	Probability	Position	Variant	Probability	Position	Variant	Probability	Position	Variant	Probability
11330005	rs62129150	1.00	11334295	rs4804155	1.00	11350086	rs115758240	1.00	11340498	rs73506650	1.00
11321705	rs8113156	1.00	11330005	rs62129150	1.00	11334295	rs4804155	1.00	11340057	rs17001244	1.00
			11327571	rs4804150	1.00	11330005	rs62129150	1.00	11334295	rs4804155	1.00
						11327571	rs4804150	1.00	11330005	rs62129150	1.00
									11327571	rs4804150	1.00

METSIM Finnish and WHI African American

2 causal variants			3 causal variants			4 causal variants			5 causal variants		
Position	Variant	Probability	Position	Variant	Probability	Position	Variant	Probability	Position	Variant	Probability
11330005	rs62129150	1.00	N/A	N/A	N/A	11334295	rs4804155	1.00	N/A	N/A	N/A
11321705	rs8113156	1.00				11333596	rs3810308	1.00			
						11330005	rs62129150	1.00			
						11327227	rs11085764	1.00			

PAINTOR analysis was performed in METSIM, WHI, and METSIM/WHI combined. In each dataset, 2, 3, 4, or 5 causal variants were assumed. Posterior probabilities are presented. Positions are on chromosome 19 and hg19. N/A, no variants are predicted.
